# Community Mitigation Guidelines to Prevent Pandemic Influenza — United States, 2017

**DOI:** 10.15585/mmwr.rr6601a1

**Published:** 2017-04-21

**Authors:** Noreen Qualls, Alexandra Levitt, Neha Kanade, Narue Wright-Jegede, Stephanie Dopson, Matthew Biggerstaff, Carrie Reed, Amra Uzicanin, CDC Community Mitigation Guidelines Work Group, Alexandra Levitt, Stephanie Dopson, Mark Frank, Rachel Holloway, Lisa Koonin, Sonja Rasmussen, Stephen Redd, Christopher de la Motte Hurst, Neha Kanade, Noreen Qualls, Jeanette Rainey, Amra Uzicanin, Matthew Biggerstaff, Daniel Jernigan, Carrie Reed

**Affiliations:** 1 Division of Global Migration and Quarantine, National Center for Emerging and Zoonotic Infectious Diseases, CDC, Atlanta, Georgia; 2 Office of Infectious Diseases, CDC, Atlanta, Georgia; 3 Eagle Medical Services, San Antonio, Texas; 4 Karna, Atlanta, Georgia; 5 Division of State and Local Readiness, Office of Public Health Preparedness and Response, CDC, Atlanta, Georgia; 6 Influenza Division, National Center for Immunization and Respiratory Diseases, CDC, Atlanta, Georgia; Office of Infectious Diseases, CDC,; Influenza Coordination Unit, Office of Infectious Diseases, CDC; Influenza Coordination Unit, Office of Infectious Diseases, CDC; Influenza Coordination Unit, Office of Infectious Diseases, CDC; Influenza Coordination Unit, Office of Infectious Diseases, CDC; Influenza Coordination Unit, Office of Infectious Diseases, CDC; Influenza Coordination Unit, Office of Infectious Diseases, CDC; Division of Global Migration and Quarantine, National Center for Emerging and Zoonotic Infectious Diseases, CDC; Division of Global Migration and Quarantine, National Center for Emerging and Zoonotic Infectious Diseases, CDC; Division of Global Migration and Quarantine, National Center for Emerging and Zoonotic Infectious Diseases, CDC; Division of Global Migration and Quarantine, National Center for Emerging and Zoonotic Infectious Diseases, CDC; Division of Global Migration and Quarantine, National Center for Emerging and Zoonotic Infectious Diseases, CDC; Influenza Division, National Center for Immunization and Respiratory Diseases; CDC; Influenza Division, National Center for Immunization and Respiratory Diseases; CDC; Influenza Division, National Center for Immunization and Respiratory Diseases; CDC.

## Abstract

When a novel influenza A virus with pandemic potential emerges, nonpharmaceutical interventions (NPIs) often are the most readily available interventions to help slow transmission of the virus in communities, which is especially important before a pandemic vaccine becomes widely available. NPIs, also known as community mitigation measures, are actions that persons and communities can take to help slow the spread of respiratory virus infections, including seasonal and pandemic influenza viruses.

These guidelines replace the 2007 *Interim Pre-pandemic Planning Guidance: Community Strategy for Pandemic Influenza Mitigation in the United States — Early, Targeted, Layered Use of Nonpharmaceutical Interventions* (https://stacks.cdc.gov/view/cdc/11425). Several elements remain unchanged from the 2007 guidance, which described recommended NPIs and the supporting rationale and key concepts for the use of these interventions during influenza pandemics. NPIs can be phased in, or layered, on the basis of pandemic severity and local transmission patterns over time. Categories of NPIs include personal protective measures for everyday use (e.g., voluntary home isolation of ill persons, respiratory etiquette, and hand hygiene); personal protective measures reserved for influenza pandemics (e.g., voluntary home quarantine of exposed household members and use of face masks in community settings when ill); community measures aimed at increasing social distancing (e.g., school closures and dismissals, social distancing in workplaces, and postponing or cancelling mass gatherings); and environmental measures (e.g., routine cleaning of frequently touched surfaces).

Several new elements have been incorporated into the 2017 guidelines. First, to support updated recommendations on the use of NPIs, the latest scientific evidence available since the influenza A (H1N1)pdm09 pandemic has been added. Second, a summary of lessons learned from the 2009 H1N1 pandemic response is presented to underscore the importance of broad and flexible prepandemic planning. Third, a new section on community engagement has been included to highlight that the timely and effective use of NPIs depends on community acceptance and active participation. Fourth, to provide new or updated pandemic assessment and planning tools, the novel influenza virus pandemic intervals tool, the Influenza Risk Assessment Tool, the Pandemic Severity Assessment Framework, and a set of prepandemic planning scenarios are described. Finally, to facilitate implementation of the updated guidelines and to assist states and localities with prepandemic planning and decision-making, this report links to six supplemental prepandemic NPI planning guides for different community settings that are available online (https://www.cdc.gov/nonpharmaceutical-interventions).

## Introduction

Nonpharmaceutical interventions (NPIs) are strategies for disease, injury, and exposure control (https://www.cdc.gov/phpr/capabilities/DSLR_capabilities_July.pdf). They include actions that persons and communities can take to help slow the spread of respiratory viruses (e.g., seasonal and pandemic influenza viruses). These actions include personal protective measures for everyday use (e.g., staying home when ill, covering coughs and sneezes, and washing hands often) and communitywide measures reserved for pandemics and aimed at reducing opportunities for exposure (e.g., coordinated closures and dismissals of child care facilities and schools and cancelling mass gatherings). When a novel influenza A virus with pandemic potential emerges, NPIs can be used in conjunction with available pharmaceutical interventions (antiviral medications) to help slow its transmission in communities, especially when a vaccine is not yet widely available. Given current vaccine technology, a pandemic vaccine might not be available for up to 6 months (https://www.fda.gov/%20ForConsumers/ConsumerUpdates/ucm336267.htm). NPIs can be used before a pandemic is declared in areas where a novel influenza A virus is detected and during a pandemic.

These 2017 guidelines provide evidence-based recommendations on the use of NPIs in mitigating the effects of pandemic influenza. These guidelines update and expand the 2007 strategy (https://stacks.cdc.gov/view/cdc/11425).*

## Purpose

The purpose of these guidelines is to help state, tribal, local, and territorial health departments with prepandemic planning and decision-making by providing updated recommendations on the use of NPIs. These recommendations have incorporated lessons learned from the federal, state, and local responses to the influenza A (H1N1)pdm09 virus pandemic (hereafter referred to as the 2009 H1N1 pandemic) and findings from research. Communities, families and individuals, employers, and schools can create plans that use these interventions to help slow the spread of a pandemic and prevent disease and death.

**FIGURE 1:**
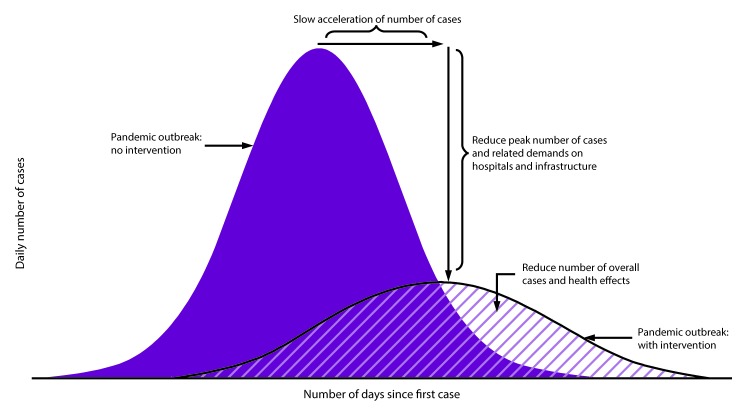
Goals of community mitigation for pandemic influenza **Source:** Adapted from: CDC. Interim pre-pandemic planning guidance: community strategy for pandemic influenza mitigation in the United States—early, targeted, layered use of nonpharmaceutical interventions. Atlanta, GA: US Department of Health and Human Services, CDC; 2007. https://stacks.cdc.gov/view/cdc/11425.

Specific goals for implementing NPIs early in a pandemic include slowing acceleration of the number of cases in a community, reducing the peak number of cases during the pandemic and related health care demands on hospitals and infrastructure, and decreasing overall cases and health effects (Figure 1). When a pandemic begins, public health authorities need to decide on an appropriate set of NPIs for implementation and to reiterate the importance of personal protective measures for everyday use (e.g., voluntary home isolation of ill persons [staying home when ill], respiratory etiquette, and hand hygiene) and environmental cleaning measures (e.g., routine cleaning of frequently touched surfaces), which are recommended at all times for prevention of respiratory illnesses (Table 1). Personal protective measures reserved for pandemics (e.g., voluntary home quarantine of exposed household members [staying home when a household member is ill] and use of face masks by ill persons) also might be recommended (Table 1). A more difficult decision is how and when to implement community-level NPIs that might be warranted but are more disruptive (e.g., temporary school closures and dismissals, social distancing in workplaces and the community, and cancellation of mass gatherings) (Table 1). These decisions are made by state and local officials on the basis of conditions in the applicable jurisdictions, with guidance from CDC (according to pandemic severity and potential efficacy) and governing authorities (*1*). Prepandemic planning, along with community engagement, is an essential component of these decisions (Table 2).

**TABLE 1:** Nonpharmaceutical interventions for personal and community preparedness to prevent pandemic influenza

NPI category*	NPIs	Timing
**Personal**		
Personal protective measures for everyday use	Voluntary home isolation of ill persons (staying home when ill)	Recommended at all times
	Respiratory etiquette	
	Hand hygiene	
Personal protective measures reserved for pandemics	Voluntary home quarantine of exposed household members (staying home for up to 3 days^†^ when a household member is ill)	Reserved for pandemics
	Use of face masks in community settings when ill	
**Community**		
School closures and dismissals^§^	Temporary, preemptive, coordinated dismissals of child care facilities and schools for grades K–12^¶^	Reserved for pandemics
Social distancing measures (examples)	Dividing classes into smaller groups and creating opportunities for distance learning (e.g., via the internet or local television or radio stations)	Reserved for pandemics
	Telecommuting and remote-meeting options in workplaces	
	Mass gathering modifications, postponements, or cancellations	
**Environmental**		
Environmental surface cleaning measures	Routine cleaning of frequently touched surfaces and objects in homes, child care facilities, schools, and workplaces	Recommended at all times

**Abbreviation:** NPI = nonpharmaceutical intervention.

*Personal, community, and environmental NPIs should be 1) initiated early in a pandemic before local epidemics begin to grow exponentially, 2) targeted toward the nexus of transmission (in affected areas where the novel virus circulates), and 3) layered together to reduce community transmission to the greatest extent possible.

^†^If the incubation period for the next pandemic influenza virus is longer or shorter than 3 days, CDC will amend the recommendation.

^§^A school closure involves closing a school and sending all the students and staff members home. A school dismissal could involve a school staying open for staff members while the students stay home.

^¶^Preemptive, coordinated dismissals might be implemented early during a pandemic to decrease the spread of influenza before many students and staff members become ill. Selective dismissals might be implemented by schools that serve students at high risk for complications from infection with influenza. Reactive dismissals might be implemented when many students and staff members are ill and not attending school or when many students and staff members are arriving at school ill and being sent home. Selective and reactive dismissals do not help slow disease transmission in the community.

**TABLE 2:** Factors to consider before implementing nonpharmaceutical interventions during an influenza pandemic

Planning factors	Planning goals	Activities
Ethical considerations	• Community engagement in prepandemic planning • Equitable distribution of public health resources during a pandemic	• Promoting public input into NPI planning • Ensuring that NPIs benefit all groups within a community • Carefully considering and justifying any restrictions on individual freedom needed to implement NPIs (e.g., voluntary home quarantine of exposed household members)
Feasibility of NPI implementation	• Minimal interruption of regular programs and activities • Selection of NPIs that are practical to implement within each community	• Identifying practical obstacles to NPI implementation and considering ways to overcome them. Examples include the following: — Educational issues (e.g., missed educational opportunities or loss of free or subsidized school meals because of school dismissals) — Financial issues (e.g., workers who cannot afford to stay home when they are ill or to care for an ill family member because they do not have paid sick leave) — Legal issues (e.g., local jurisdictions that do not have the legal authority to close schools or cancel mass gatherings for public health reasons) — Workplace issues (e.g., access to clean water, soap, or hand sanitizer and flexible workplace policies or arrangements)
Activation triggers, layering, and duration of NPIs	• Optimal implementation of NPIs during a pandemic	• Maximizing the effectiveness of NPIs by taking the following actions: — Identifying activation triggers to ensure early implementation of NPIs before explosive growth of the pandemic — Planning for simultaneous use of multiple NPIs because each NPI is only partially effective — Planning for long-term duration of school dismissals and social distancing measures
Selecting NPIs for groups at risk for severe influenza complications and for those with limited access to care and services	• Protection of persons most at risk for severe illness or death during a pandemic • Protection of persons who might need additional assistance during a pandemic response, including persons with disabilities and other access and functional needs	• Identifying strategies for implementing NPIs among groups at high risk for severe influenza-related complications, including the following: — Pregnant women — Persons aged <5 yrs and ≥65 yrs — Persons with underlying chronic diseases — Persons in institutions • Identifying strategies for implementing NPIs among groups who might experience barriers to or difficulties with accessing or receiving medical care and services, including the following: — Persons who are culturally, geographically, or socially isolated or economically disadvantaged — Persons with physical disabilities, limitations, or impairments — Persons with low incomes, single-parent families, and residents of public housing — Persons who live in medically underserved communities
Public acceptance of NPIs	• Active participation in NPI implementation during a pandemic	• Promoting public understanding that individual action is essential for effective implementation of NPIs in every pandemic scenario. In many scenarios, both personal and community NPIs might be recommended. NPI recommendations might change as new knowledge is gained. • Identifying key personnel to disseminate emergency information (e.g., alerts, warnings, and notifications) and establishing communication channels that enable members of the public to ask questions and express concerns (e.g., call centers or social media sites) • Ensuring that school dismissals and other NPIs are acceptable to the community during a pandemic • Coordinating with local partners to support households complying with voluntary home quarantine (e.g., providing necessary food and supplies) • Identifying strategies for mitigating the secondary consequences of school dismissals and other social distancing measures (e.g., modifications or cancellations of mass gatherings) • Minimizing intervention fatigue* during a pandemic
Balancing public health benefits and social costs	• Maximization of NPI public health benefits and minimization of social and economic costs during a pandemic	• Estimating economic and social costs of NPIs and their secondary (unintended or unwanted) consequences • Balancing those costs against public health benefits, with reference to different prepandemic planning scenarios • Identifying strategies for reducing the cost of NPI implementation
Monitoring and evaluation of NPIs	• Ongoing guidance during a pandemic on optimal NPI implementation, maintenance, and discontinuation	• Identifying ways to monitor and evaluate the following: — Degree of transmission and severity of the evolving pandemic — Type and degree of NPI implementation — Level of compliance with NPI measures and the emergence of intervention fatigue — Effectiveness of NPIs in mitigating pandemic impact — Secondary consequences of NPIs and the effectiveness of strategies to mitigate them

**Abbreviation:** NPI = nonpharmaceutical intervention.

**Source:** Adapted from: Barrios LC, Koonin LM, Kohl KS, Cetron M. Selecting nonpharmaceutical strategies to minimize influenza spread: the 2009 influenza A (H1N1) pandemic and beyond. Public Health Rep 2012;127:565–71.

*Fatigue that results from being requested, often repeatedly, to change daily behaviors for the good of the community, especially when those changes disrupt daily life (e.g., caring for children when schools are dismissed for several weeks or avoiding crowded settings) (**Source:** Ryan JR, ed. Pandemic influenza: emergency planning and community preparedness. 2008. Boca Raton, FL: CRC Press; 2008:158).

The decision regarding whether and when to recommend additional NPIs is another component (Table 3). State and local public health departments might use certain influenza surveillance indicators to help decide when to consider implementing NPIs such as school closures and dismissals and other social distancing measures in schools, workplaces, and public settings during an influenza pandemic. The choice of influenza surveillance indicators might differ among states and localities, depending on the availability and capacity of their public health resources. Examples of possible influenza surveillance indicators include additional patient visits to health care providers for influenza-like illness (ILI) and increased geographic spread of influenza within a state. Indicators for school closures and dismissals might include increased school absenteeism rates or the earliest laboratory-confirmed influenza cases among students, teachers, or staff members. Indicators that might help confirm that NPI implementation should continue include increased influenza-associated hospitalizations or increases in adult or pediatric deaths attributed to influenza. Additional information about NPI prepandemic planning is available (supplementary Chapter 1 https://stacks.cdc.gov/view/cdc/44313).

**TABLE 3:** Examples of possible nonpharmaceutical intervention surveillance indicators for an influenza pandemic

Key influenza indicator	U.S. data source	Measure of influenza activity
**Indicators of spread or level of influenza activity**		
Percentage of patient visits to health care providers for ILI in the United States	Outpatient ILI Surveillance Network (ILINet), which includes approximately 2,900 enrolled outpatient health care providers in 50 states	Current ILI level in relation to most recent national and region-specific baseline levels, with CDC providing baseline values for the 10 HHS surveillance regions and for the United States as a whole https://www.cdc.gov/flu/weekly/overview.htm
ILI activity by state: percentage of outpatient visits for ILI in a state (ranges from minimal to high)	Outpatient ILI Surveillance Network (ILINet) Additional: Flu Near You https://flunearyou.org/	Ten activity levels that compare the mean reported percent of visits due to ILI for the current week to noninfluenza weeks, specifying the number of standard deviations at or above the mean for the current week https://www.cdc.gov/flu/weekly/FluViewInteractive.htm
Geographic spread of influenza in a state (ranges from none to widespread)	State and Territorial Epidemiologists reports	Estimated weekly levels of geographic spread (local, regional, or widespread) of influenza activity reported by state health departments https://www.cdc.gov/flu/weekly/overview.htm
Percentage of respiratory specimens that test positive for influenza viruses in the United States	Approximately 110 U.S. WHO collaborating laboratories and 240 National Respiratory and Enteric Virus Surveillance System laboratories	National and regional percentage of respiratory specimens testing positive for influenza viruses https://www.cdc.gov/flu/weekly/FluViewInteractive.htm
Absenteeism rates due to ILI in child care facilities, K–12 schools, or colleges and universities (reflects number of ILI cases)	ILI monitoring/surveillance systems in child care facilities, K–12 schools, or colleges and universities	Increased absenteeism rates due to ILI in child care facilities, K-12 schools, or colleges and universities (reflects increased number of ILI cases)
Laboratory-confirmed influenza cases among students, teachers, and staff members		Increases in laboratory-confirmed influenza cases among students, teachers, and staff members
		Laboratory-confirmed outbreaks of influenza in child care facilities, K–12 schools, or colleges and universities
**Indicators of clinical severity of influenza**		
Influenza-associated hospitalizations	Influenza Hospitalization Surveillance Network (FluSurv-NET), which collects data from the 10 Emerging Infections Program sites, as well as Michigan, Ohio, and Utah (https://wwwnc.cdc.gov/eid/article/21/9/14-1912_article)	Population-based rate of influenza-associated hospitalizations in multiple geographic areas https://www.cdc.gov/flu/weekly/FluViewInteractive.htm
Percentage of deaths attributed to pneumonia and influenza	National Center for Health Statistics mortality surveillance system	The percentage of death certificates indicating pneumonia and influenza compared with a seasonal baseline and epidemic threshold value calculated for each week (using a periodic regression model) https://www.cdc.gov/flu/weekly
Influenza-associated deaths among persons aged <18 yrs	Influenza-Associated Pediatric Mortality Surveillance System	Any laboratory-confirmed influenza-associated deaths in children, all of which are reported through this system https://www.cdc.gov/flu/weekly/FluViewInteractive.htm

**Abbreviations:** HHS = U.S. Department of Health and Human Services; ILI = influenza-like illness; WHO = World Health Organization.

## Background

An influenza pandemic occurs when a novel virus emerges for which the majority of the population has little or no immunity. Influenza pandemics are facilitated by sustained human-to-human transmission, and the infection spreads worldwide over a relatively short period (*2*). The first influenza pandemic of the 21st century began in 2009, 2 years after the 2007 strategy for prepandemic planning was published. Lessons learned during the response to the 2009 H1N1 pandemic underscored the importance of a flexible approach to the use of NPIs, particularly during the early stages of a pandemic, and led to the development of new tools for assessing pandemic severity and prepandemic planning (Box 1).

BOX 1 **Lessons learned from the 2009 H1N1 pandemic response**

The 2009 H1N1 pandemic demonstrated the unpredictable nature of influenza viruses and showed that prepandemic planning must be broad and flexible. Lessons learned during the 2009 H1N1 pandemic response from the United States and other affected countries follow.

**H1N1 and children**

The epidemiology of pandemic influenza might be different from the epidemiology of seasonal influenza; therefore, different populations might be disproportionately affected.

- An estimated 43–89 million people in the United States were infected with H1N1pdm09 virus during April 2009–April 2010, and approximately 12,000 people died (*3*).
- Severe outcomes of influenza include complications that require hospitalization and can be fatal (e.g., pneumonia or bronchitis). Severe outcomes from H1N1pdm09 virus infection were most common among children, young adults, and specific groups at high risk for complications (e.g., pregnant women) rather than in adults aged ≥65 years, the group most at risk from seasonal influenza (*4*–*7*). Over the course of the pandemic, an estimated 86,000 children were hospitalized in the United States, which is 2–3 times the number admitted during a typical influenza season (*5*). The number of deaths among children also was more than twice as high as during a regular influenza season.
- On August 28, 2009, the Advisory Committee on Immunization Practices recommended that children be placed higher on the priority list for receiving the monovalent H1N1 vaccine, which became available in October 2009 (*8*).
- Children at risk for severe outcomes from the H1N1pdm09 virus (and from any influenza virus) included those with underlying health conditions such as asthma, diabetes, obesity, or heart, lung, or neurologic diseases. Approximately 60% of hospitalized children had one or more of these conditions, compared with 80% of hospitalized adults (*5*). Infants born to mothers infected with the H1N1pdm09 virus also might have been at risk, as suggested by U.S. and Canadian studies which found that infants whose mothers received the H1N1 vaccine were less likely to be small for their gestational age or delivered preterm (*9*,*10*).

**Public health tools to assess pandemic severity and guide NPI selection**

The 2007 Pandemic Severity Index had limited usefulness because attack rates and case-fatality ratios were difficult to measure and imprecise early in the pandemic.

- The earliest available data on attack rates and case-fatality ratios suggested that the 2009 H1N1 pandemic virus was highly transmissible and caused severe outcomes. However, the cases being reported overestimated severity because they were primarily derived from mortality data.
- By May 1, 2009, which was 5 days after the U.S. Department of Health and Human Services (HHS) declared a nationwide public health emergency, CDC had received reports of 141 laboratory-confirmed H1N1pdm09 cases in 19 states, with one death in Texas (https://www.cdc.gov/h1n1flu/updates/050109.htm). On the basis of this initial information and continued reports of cases of disease with severe outcomes in Mexico, including deaths among previously healthy young adults (*11*), CDC recommended that communities with laboratory-confirmed cases of H1N1pdm09 virus consider closing child care facilities and schools, depending on the extent and severity of illness (*12*). CDC also recommended other NPIs described in the 2007 strategy, including voluntary home isolation for ill persons (i.e., staying home when ill) and voluntary home quarantine for exposed household members (i.e., staying home when a household member is ill).
- Within 12 days of recognition of the emerging pandemic, the national influenza surveillance system generated sufficient data for a refined assessment.
  - From April 23, 2009, when H1N1pdm09 virus was detected in California (*13*), through May 5, 2009, CDC received reports of 403 confirmed cases of H1N1pdm09 virus in 38 states. The low rates of hospitalizations and deaths, as well as reported attack rates similar to those for seasonal influenza, suggested that the majority of U.S. cases were less severe than those reported from Mexico.
  - CDC issued new nonpharmaceutical intervention (NPI) guidance on May 5, 2009 (*14*), recommending that although ill students and teachers should stay home, schools did not need to close. The guidance acknowledged that public health authorities in certain jurisdictions might still decide to close schools on the basis of local considerations, including public concern, school absenteeism, and staffing shortages.
  - During August–December 2009, communities in 46 U.S. states implemented 812 dismissal events (i.e., a single school dismissal or dismissal of all schools in a district), affecting 1,947 schools with approximately 623,616 students and 40,521 teachers (*15*). The 1,947 schools included 639 urban and 1,250 rural schools, representing 0.7% and 3.3% of all urban and rural schools, respectively, in the United States.
- The recognition that the Pandemic Severity Index was of limited use during the earliest stages of an actual pandemic led to the development of a new tool for evaluating the potential effects of an emerging pandemic, the Pandemic Severity Assessment Framework (PSAF) (supplementary Chapter 2 https://stacks.cdc.gov/view/cdc/44313).

**NPIs and influenza transmission**

Well-established methods to prevent seasonal influenza transmission, such as hand hygiene promotion, also were effective in pandemic influenza settings to prevent the spread of H1N1pdm09 virus in some communities.

- **Hand hygiene.** A randomized trial, conducted over 12 weeks in 60 elementary schools in Cairo, Egypt, during the 2009 H1N1 pandemic, demonstrated a 47% reduction in confirmed cases of influenza after twice-daily hand washing and health hygiene instruction in comparison with a control group that did not receive health hygiene instruction or have access to soap and hand-drying materials (*16*). This study demonstrated the effects of hand washing on laboratory-confirmed influenza in a population of persons that typically have little or no access to soap or hand-drying materials and among whom frequent hand washing is not standard.
- **School closures and dismissals.** Data from the United States, Canada, and Mexico suggest that early implementation of school closures and dismissals reduced the spread of H1N1pdm09 virus.
  - Two waves of the 2009 H1N1 pandemic occurred in the United States, one in spring 2009 and one in fall 2009. The majority of pandemic cases occurred during the fall wave (*4*), as H1N1pdm09 cases surged in many U.S. communities about 2 weeks after schools reopened after summer break. Opening dates for schools ranged from early August through early September (*17*). A comparison of Texas school districts that closed versus those that stayed open during the pandemic found that school closure was associated with a 45%–72% reduction in acute respiratory illness in households with school-aged children (*18*).
  - Mathematical models suggested that school closures in Alberta, Canada, in May 2009 were associated with reduced transmission among school children by approximately 50%, attenuating the first wave of the 2009 H1N1 epidemic (*19*).
  - H1N1pdm09 virus transmission in the greater Mexico City, Mexico, area decreased by an estimated 29%–37% after school closures and implementation of other social distancing measures (*20*).
  - After conducting a systematic review of scientific literature published through February 2011, including initial data gathered during the 2009 H1N1 pandemic, the U.S. Community Preventive Services Task Force found insufficient evidence to determine whether the public health benefits of preemptive, coordinated school dismissals balanced their economic and social costs during a mild or moderate influenza pandemic. However, the task force did recommend preemptive, coordinated school dismissals during a severe pandemic (*21*).
- **Social distancing measures.** H1N1pdm09 virus transmission in Mexico decreased significantly after school closures and implementation of other social distancing measures (*20*,*22*). In the United States, schools in Georgia that shortened school days had less absenteeism due to severe respiratory illness (*23*).

Additional assessments are needed to determine the value of combining voluntary home quarantine with antiviral chemoprophylaxis.

- Although the 2007 strategy suggested that communities consider combining voluntary home quarantine with prophylactic use of antiviral medications, assuming a feasible means of distribution, HHS did not adopt antiviral chemoprophylaxis as its official policy because of concerns about insufficient supplies and drug resistance.
- During the 2009 H1N1 pandemic, antiviral chemoprophylaxis of exposed persons contained the spread of the disease, along with the implementation of social distancing measures, in a few small, well-defined settings, including a summer camp (*24*) and a cruise ship (*25*). Moreover, an observational cohort study of 259 households in the United Kingdom found that administration of antiviral medications to 285 confirmed patients and their 761 close contacts was very effective (92%) in preventing household transmission (*26*).
- Although limited, the H1N1pdm09 experience suggests that antiviral chemoprophylaxis might be recommended in the future in some settings as an adjunct to self-quarantine, assuming that additional antiviral medications are on the market, providing more treatment choices and making the emergence of drug resistance less of a concern. However, this recommendation would require much greater quantities of antiviral medications, even if no new products are developed, to ensure sufficient supplies.

**Mobilizing the public**

Most members of the public complied with public health recommendations regarding hand hygiene and social distancing.

- The Harvard Opinion Research Program conducted 13 polls on the response of the U.S. public during the 2009–2010 pandemic, including the response of the general public, pregnant women, new mothers, parents, and businesses. These randomized telephone polls found the following:
  - A total of 59% of 1,067 Americans reported washing their hands or using hand sanitizer more frequently during the 2009 H1N1 pandemic (*27*). A total of 25% avoided places where numerous people tend to gather, such as sporting events, malls, or public transportation.
  - Most (85%) of 514 pregnant women washed or sanitized their hands more frequently to reduce the chance of infection with H1N1pdm09 virus (*27*). A total of 68% reported taking steps to avoid proximity to someone who had influenza-like symptoms, and 31% avoided mass gathering places. Most (91%) of 526 new mothers also washed or sanitized their hands more frequently, and 81% took steps to avoid being near someone who had influenza-like symptoms.

School-related NPIs, including school closures and dismissals, were acceptable and feasible.

- According to a Harvard Opinion Research Program poll of 523 parents from 39 U.S. states whose child care center or school closed temporarily in response to the 2009 H1N1 pandemic, 90% of parents agreed with the dismissal decision, and 85% believed the dismissal reduced influenza transmission (*27*,*28*).
  - A total of 75% of parents who responded stated that the dismissal was not a problem, and 3% stated it was a major problem. Approximately 20% of parents reported that an adult in the household missed work because of the dismissal, and 19% had a child who missed a free or reduced-cost lunch. Of these, 2% and <1%, respectively, said missing work and missing lunch were major problems.
  - Most of the 523 parents polled believed that at least one of the following factors was a major reason the institution had closed: 1) to keep children apart and reduce the chance they would infect each other (81%), 2) because the school decided cleaning the building and surfaces that children touch was important for reducing the spread of the illness (73%), and 3) because the school or child care center could not operate effectively when numerous students were absent (58%).
- A study conducted through an online survey of school principals showed that implementing NPIs in public schools in New York City, New York, was feasible during the 2009 H1N1 pandemic (*29*). Schools successfully implemented respiratory etiquette education, hand-hygiene measures, and environmental measures and isolated ill students. Another online survey found that the majority of public schools in Georgia also were able to successfully implement both personal and community NPIs recommended by CDC (*23*).

Public health practitioners should be prepared to explain that the initial pandemic guidance might change if a pandemic is more or less severe than initially assessed.

- Within 12 days of recognizing the emerging pandemic on April 23, 2009, CDC updated its initial guidance on NPIs (issued on May 1, 2009) on May 5, 2009, on the basis of more complete and robust data that suggested that the majority of U.S. cases were less severe than those reported from Mexico.
- Certain public health departments reported difficulties in communicating the updated guidance on school closures to their communities, especially communities that were planning to implement school closures or had already done so (*30*,*31*).
- The H1N1pdm09 experience with school closures suggests the need to coordinate and harmonize school closure policies across jurisdictions and to proactively communicate and explain any jurisdictional differences.

Public engagement, community preparedness, and trust in government action are important for successful NPI implementation during a pandemic.

- Practical obstacles to NPI implementation that required community-level solutions included 1) ill persons going to work because they lacked unpaid leave (*32*), 2) lack of clarity about decision-making authority to close schools for public health reasons in some jurisdictions (*30*,*33*), and 3) lack of access to clean water, soap, or hand sanitizer in some workplaces.
- Although 74% of 1,057 businesses that participated in a Harvard Opinion Research Program poll on business preparedness for the H1N1pdm09 virus offered paid sick leave for at least some workers (*27*), fewer offered paid leave that would allow workers to take care of ill family members (35%) or to take time off to care for children if schools or child care centers closed (21%).
- In large cities such as New York City, New York, rapid implementation of local-level response strategies required advanced planning and preparation, as well as high-level political leadership; collaboration between public health and emergency management agencies; coordination with businesses, nongovernmental organizations, and community- and faith-based organizations; and transparent communication with the public (*34*).
- During future pandemics, local health policies and risk communication strategies should take into account community attitudes and acceptance of preventive behaviors related to social distancing, hand hygiene, and vaccination, which might differ across racial and economic groups (*35*).
- According to an online survey of a nationally representative sample conducted by the University of Maryland, clear and consistent communication by public health authorities and government spokespersons, including the use of role models, was important to the public’s trust in government actions during the 2009 H1N1 pandemic (*36*). Although the University of Maryland study focused on risk communications related to H1N1 vaccination, this finding also is likely to apply to public attitudes about NPI implementation.

### Lessons Learned from the 2009 H1N1 Pandemic Response

The 2009 H1N1 pandemic was a reminder to be prepared for the unpredictable nature of pandemics. Knowing in advance which subtype of pandemic virus will emerge is impossible, as is where and when it will emerge, how quickly the virus will spread, how severe the illness will be, and who will be the most affected. Because of this unpredictability, prepandemic planning must be broad and flexible.

The 2007 strategy for prepandemic planning was developed with the assumption that the next influenza pandemic would be severe, like the 1957 pandemic, which was characterized by high transmissibility and medium clinical severity. When the 2007 strategy was developed, the primary concern was that a pandemic virus might evolve from the highly pathogenic avian influenza A (H5N1) virus, a virus that reemerged in Asia in 2003 in domestic poultry and spread to Africa, the Middle East, and Europe among poultry, with sporadic zoonotic transmission (*37*). Moreover, CDC thought that this virus would most likely emerge overseas, providing the United States with time to prepare for a domestic response, including making use of prepandemic H5N1 vaccine in CDC’s Strategic National Stockpile. Instead, the 2009 pandemic influenza A virus turned out to be a novel H1N1 virus that appears to have emerged in southern Mexico and was first identified in two persons in California (*13*). Although the 2009 H1N1 pandemic in the United States was moderate in terms of overall morbidity and mortality among the U.S. general population, severe outcomes from H1N1pdm09 virus infection were more common among children, young adults, and specific groups at risk for serious complications (e.g., pregnant women) than among older adults (Box 1).

Although the emergence of the H1N1pdm09 virus prompted development of pandemic vaccines, a pandemic vaccine was not available until October 2009, 6 months after the initial report that identified the pandemic virus. In addition, another 2 months were required (December 2009) for sufficient stocks to be manufactured, distributed, and available to vaccinate several population groups, including school-aged children and persons living with or caring for infants aged <6 months, as recommended by the Advisory Committee on Immunization Practices (ACIP).^†^ Even though work is ongoing to accelerate the pace of development, distribution, and administration of a vaccine during future pandemics, this experience reaffirmed the importance of the use of NPIs in the early stages of a pandemic before a well-matched vaccine is widely available (i.e., vaccines produced using a virus that is very similar to the circulating virus).

Another lesson learned about NPI implementation during the 2009 H1N1 pandemic was that rapidly changing guidance can create confusion and difficulties during implementation (Box 1) (*30*,*31*). Nevertheless, field studies found that school-related NPIs, including school closures recommended to mitigate the impact of the 2009 H1N1 pandemic during spring 2009, were considered acceptable and feasible for most parents and caregivers, even when parents had to miss work and in the absence of free or reduced-cost school lunches for students (*28*,*38*–*41*). Other interventions that reduced the spread of H1N1pdm09 virus in some communities included hand hygiene (*42*), regularly scheduled school summer breaks (*19*), and social distancing measures, such as cancelling mass gatherings and closing public places (*22*).

### Community Engagement

The 2009 H1N1 pandemic underscored that effective prepandemic planning requires the involvement of public health and local leaders, employers, organizations, and stakeholders and is essential to ensure timely and effective use of NPIs to limit disease spread during a pandemic (Box 2). Effective use of NPIs depends on the acceptance and participation of individual persons who implement personal protective measures and of communities that implement communitywide measures such as temporary school closures (https://www.cdc.gov/phpr/capabilities/DSLR_capabilities_July.pdf).

BOX 2 **Principles of community engagement**

**Planning**

Before initiating a community engagement effort, consider the following:

1. Be clear about the purpose or goals of the engagement effort and the relevant populations and communities.

2. Become knowledgeable about the community’s culture, economic conditions, social networks, political and power structures, norms and values, demographic trends, history, and experience with efforts by outside groups to engage it in various programs. Learn about the community’s perceptions of those initiating the engagement activities.

**Initiation**

For engagement to occur, the following steps are necessary:

3. Go to the community, establish relationships, build trust, work with the formal and informal leaders, and seek commitment from community organizations and leaders to create processes for mobilizing the community.

4. Remember and accept that collective self-determination is the responsibility and right of all people in a community. No external entity should assume the ability to bestow on a community the power to act in its own self-interest.

**Implementation**

For engagement to succeed, consider the following:

5. Partnering with the community is necessary to create change and improve health.

6. All aspects of community engagement must recognize and respect the diversity of the community. Awareness of the various cultures of a community and other factors affecting diversity must be paramount in planning, designing, and implementing approaches to engaging a community.

7. Community engagement can only be sustained by identifying and mobilizing community assets and strengths and by developing the community’s capacity and resources to make decisions and take action.

8. Organizations that would like to involve a community and those seeking to effect change must be prepared to release control of actions or interventions to the community and be flexible enough to meet changing needs.

9. Community collaboration requires long-term commitment by the engaging organizations and its partners.

**Source:** Adapted from: Agency for Toxic Substances and Disease Registry. Principles of community engagement. Atlanta, GA: CDC, Agency for Toxic Substances and Disease Registry. https://www.atsdr.cdc.gov/communityengagement/pdf/PCE_Report_Chapter_2_SHEF.pdf

The 2007 guidance took into account the results of a 2006 opinion poll conducted with a representative national sample of 1,697 adults aged ≥18 years. The results indicated that when faced with an outbreak of pandemic influenza, the majority of persons in the United States would be willing to make major changes in their lives and cooperate with public health recommendations on the use of NPIs (http://archive.sph.harvard.edu/press-releases/2006-releases/press10262006.html). Findings were similar in a follow-up study during the 2009–2010 H1N1 pandemic (Box 1) (https://www.hsph.harvard.edu/horp/project-on-the-public-response-to-h1n1).

For example, in 2006, 85% of the respondents said that they and all members of their household would stay home for 7–10 days if another household member were ill with pandemic influenza. The H1N1 opinion polls also identified barriers to implementation of NPIs among persons and communities (e.g., the ability to stay home when ill, job security, and income protection) (https://www.hsph.harvard.edu/horp/project-on-the-public-response-to-h1n1). States and localities could establish local planning councils or hold public engagement meetings that address these and other issues related to public health preparedness, pandemic education, and planning. States and local communities also can draw on planning guidance provided in the CDC Public Health Preparedness Capabilities: National Standards for State and Local Planning, which lists NPIs as one of 15 capabilities (https://www.cdc.gov/phpr/capabilities/DSLR_capabilities_July.pdf). Additional information about pandemic influenza and NPI community engagement is available (supplementary Chapter 1 https://stacks.cdc.gov/view/cdc/44313).

### New Tools for Prepandemic Planning and Pandemic Assessment

#### Novel Influenza Virus Pandemic Intervals

**FIGURE 2:**
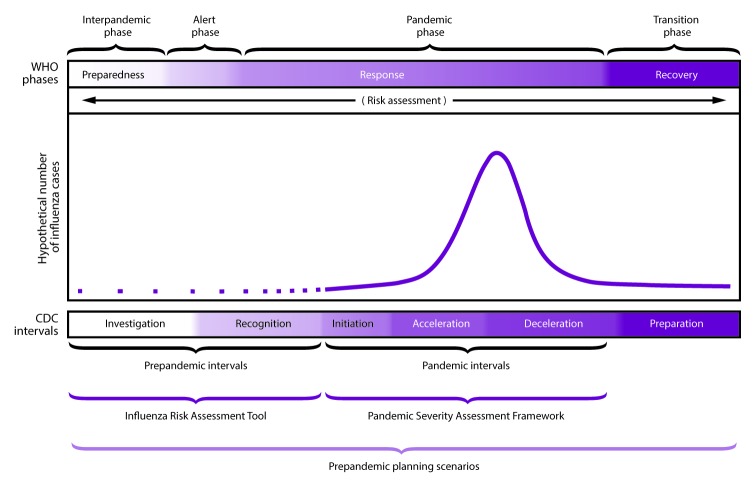
Preparedness and response framework for novel influenza A virus pandemics, with CDC intervals and World Health Organization phases **Source:** Adapted from: Holloway R, Rasmussen SA, Zaza S, Cox NJ, Jernigan DB; Influenza Pandemic Framework Workgroup. Updated preparedness and response framework for influenza pandemics. MMWR Recomm Rep 2014;63(No. RR-6).

In 2014, CDC updated its 2008 guidance on pandemic intervals to include six intervals that describe influenza pandemic progression in a way that supports flexible prepandemic preparedness and response. The intervals include 1) investigation of novel influenza cases, 2) recognition of potential for ongoing transmission, 3) initiation, 4) acceleration, 5) deceleration of the pandemic wave, and 6) preparation for a future pandemic wave (*43*). These intervals can be used during prepandemic planning and can serve as a platform for public health decision-making and actions during the beginning of a potential influenza pandemic. Each interval is associated with particular response activities, including implementation of select NPIs during the initiation and acceleration intervals and coordinated discontinuation of select community-level NPIs reserved for pandemics during the deceleration interval (Figure 2) (Table 4). Although the six-interval framework describes the sequence of pandemic disease evolution over time, the framework does not characterize the transmissibility of the virus or the clinical severity of the outbreak. Therefore, CDC has developed additional tools for pandemic planning and response, including the Influenza Risk Assessment Tool (supplementary Chapter 2 https://stacks.cdc.gov/view/cdc/44313); https://www.cdc.gov/flu/pandemic-resources/tools/risk-assessment.htm) and the Pandemic Severity Assessment Framework (PSAF). Additional information about the pandemic intervals is available (supplementary Chapter 2 https://stacks.cdc.gov/view/cdc/44313).

**TABLE 4:** CDC novel influenza virus pandemic intervals

Intervals	Indicators
**Investigation:** Investigation of novel influenza cases	This interval is indicated by the identification of an animal case of influenza A subtype with potential implications for human health or identification of a human case of novel influenza A anywhere in the world.
**Recognition:** Recognition of potential for ongoing transmission	This interval is indicated by an increasing number of cases or clusters of novel influenza A in humans and by virus characteristics indicating potential for ongoing human-to-human transmission anywhere in the world.
**Initiation:** Initiation of the pandemic wave	This interval is indicated by confirmation of cases of novel influenza A in humans and demonstration of efficient and sustained human-to-human transmission anywhere in the world.
**Acceleration:** Acceleration of the pandemic wave	This interval is indicated by an increasing rate of novel influenza A cases identified nationally, indicating establishment in the country.
**Deceleration:** Deceleration of the pandemic wave	This interval is indicated by decreasing rates of novel influenza A infection.
**Preparation:** Preparation for a future pandemic wave	This interval is indicated by sporadic cases of novel influenza A infection and surveillance rates returning to baseline.

**Source:** Holloway R, Rasmussen SA, Zaza S, Cox NJ, Jernigan DB; Influenza Pandemic Framework Workgroup. Updated preparedness and response framework for influenza pandemics. MMWR Recomm Rep 2014;63(No. RR-6).

#### Pandemic Severity Assessment Framework

An influenza pandemic can range from mild to extremely severe in terms of clinical severity and transmission rate. When a pandemic emerges, public health authorities should assess its projected impact and recommend rapid action to reduce virus transmission, protect populations at high risk for complications, and minimize societal disruption. As observed during the 2009 H1N1 pandemic response, attack rates and case-fatality ratios can be difficult to measure early in a pandemic because of variations in care-seeking behavior and testing practices; not everyone seeks care for their illness, and not everyone is tested and receives a diagnosis of pandemic influenza. As a result, severe cases might be more likely to be reported, resulting in an overestimate of the case-hospitalization or case-fatality ratio. Tools for prepandemic planning have been updated and augmented based on that experience, and the Pandemic Severity Index in the 2007 guidance has been replaced with PSAF. PSAF uses multiple clinical and epidemiologic indicators to provide a more comprehensive assessment of the transmissibility and clinical severity of an emerging pandemic. Whereas the Pandemic Severity Index was based on the assumption that a future pandemic would cause an illness rate of 30% in the U.S. population and relied on an assessment of case-fatality ratios to determine severity of an evolving pandemic, PSAF incorporates multiple measures of clinical severity (e.g., case-fatality ratios, case-hospitalization ratios, and deaths-hospitalizations ratios) and viral transmissibility (e.g., secondary household attack rates, school attack rates, workplace attack rates, community attack rates, or all of these, as well as rates of emergency department and outpatient visits for ILI) (*44*).

**FIGURE 3:**
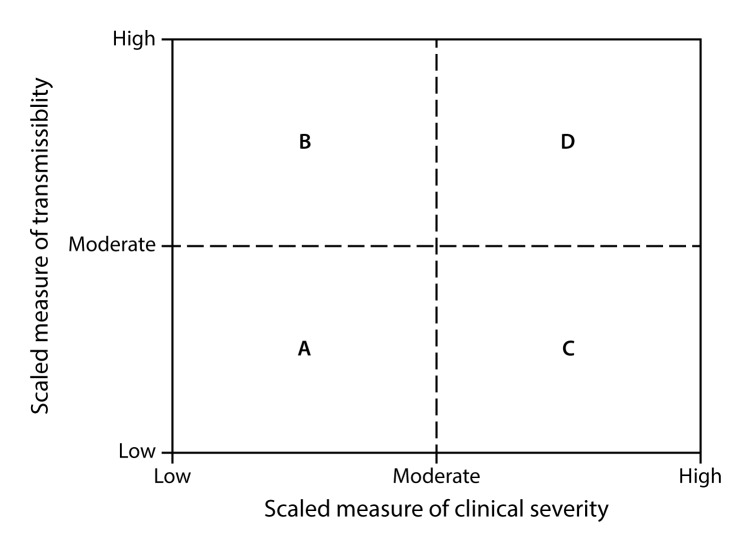
Pandemic Severity Assessment Framework for the initial assessment of the potential impact of an influenza pandemic **Source:** Reed C, Biggerstaff M, Finelli L, et al. Novel framework for assessing epidemiologic effects of influenza epidemics and pandemics. Emerg Infect Dis 2013;19:85–91.

**FIGURE 4:**
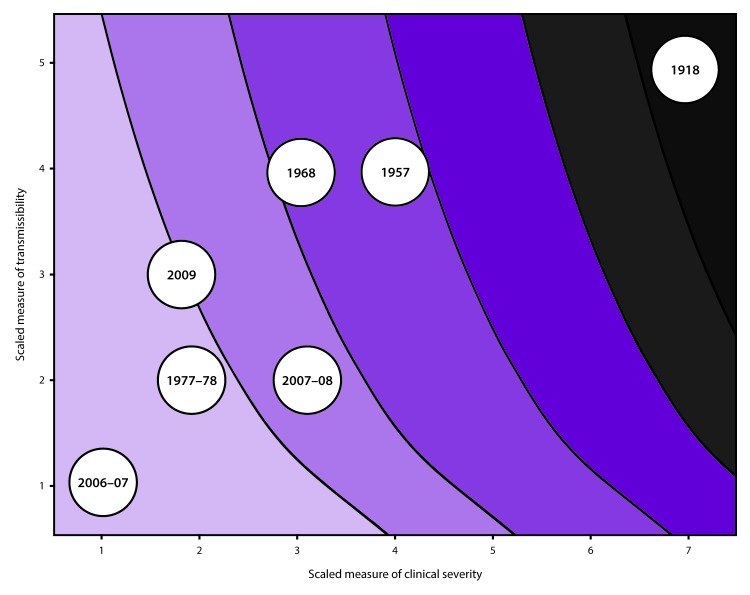
Pandemic Severity Assessment Framework using surveillance indicators for the refined assessment* of an influenza pandemic on the basis of past pandemics and influenza seasons **Source: **Adapted from: Reed C, Biggerstaff M, Finelli L, et al. Novel framework for assessing epidemiologic effects of influenza epidemics and pandemics. Emerg Infect Dis 2013;19:85–91. * Colors transition from light to dark as the estimated number of deaths increases. Transmissibility: measured on a scale of 1–5 and includes school, workplace, and community attack rates, secondary household attack rates, school and/or workplace absenteeism rates, and rates of emergency department and outpatient visits for influenza-like illness. Clinical severity: measured on a scale of 1–7 and includes case-fatality ratios, case-hospitalization ratios, and deaths-hospitalizations ratios.

When a pandemic begins, in the United States or anywhere in the world, CDC makes an initial assessment of viral transmissibility and clinical severity on the basis of these multiple PSAF measures (Table 5) (*44*). On the basis of the initial assessment, CDC recommends that affected U.S. jurisdictions respond (and other jurisdictions prepare to respond). Although data are limited during the initial 3–4 weeks after the emergence of a pandemic virus, these early data are compiled into a broad, preliminary assessment. CDC uses PSAF scores of viral transmissibility and clinical severity to place the pandemic within one of four assessment quadrants (Figure 3). Depending on the surveillance capacity in the location where the novel virus emerges and first spreads, 4–8 weeks or longer might be required to accrue sufficient data for a refined assessment of an evolving pandemic. Once data are available, the refined assessment is used to more precisely characterize the clinical severity and transmissibility of the pandemic virus (Figure 4) (Table 6). These initial and refined assessments of pandemic severity are used, in coordination with state and local public health partners, to guide the use of NPI measures. Additional information about PSAF is available (supplementary Chapter 2 https://stacks.cdc.gov/view/cdc/44313).

**TABLE 5:** Initial assessment: scaled measures of influenza virus transmissibility and clinical severity

Measures of transmissibility and clinical severity	Scale	
	Low to moderate	Moderate to high
Transmissibility		
**Secondary attack rate, household**	**≤20%**	**>20%**
**Attack rate, school or university**	≤30%	>30%
**Attack rate, workplace or community**	≤20%	>20%
**R_0_: basic reproductive number**	1–1.7	≥1.8
**Underlying population immunity**	Some underlying population immunity	Little to no underlying population immunity
**Emergency department or other outpatient visits for influenza-like illness**	<10%	≥10%
**Virologic characterization**	Genetic markers for transmissibility absent	Genetic markers for transmissibility present
**Animal models, transmission studies**	Less efficient or similar to seasonal influenza	More efficient than seasonal influenza
**Clinical severity**		
Upper bound of case-fatality ratio	<1%	≥1%
Upper bound of case-hospitalization ratio	<10%	≥10%
Deaths-hospitalizations ratio	<10%	≥10%
Virologic characterization	Genetic markers for virulence absent	Genetic markers for virulence present
Animal models, evaluation of morbidity and mortality	Less virulent or similar to seasonal influenza	More virulent than seasonal influenza

**Source:** Reed C, Biggerstaff M, Finelli L, et al. Novel framework for assessing epidemiologic effects of influenza epidemics and pandemics. Emerg Infect Dis 2013;19:85–91.

**TABLE 6:** Refined assessment: scaled measures of influenza virus transmissibility and clinical severity

Measures of transmissibility and clinical severity	Scale

1	2	3	4	5	6	7
**Transmissibility (scale of 1–5)**							
Symptomatic attack rate, community	≤10%	11%–15%	16%–20%	21%–24%	≥25%	—	—
Symptomatic attack rate, school	≤20%	21%–25%	26%–30%	31%–35%	≥36%	—	—
Symptomatic attack rate, workplace	≤10%	11%–15%	16%–20%	21%–24%	≥25%	—	—
Household secondary attack rate, symptomatic	≤5%	6%–10%	11%–15%	16%–20%	≥21%	—	—
R0: basic reproductive number	≤1.1	1.2–1.3	1.4–1.5	1.6–1.7	≥1.8	—	—
Peak percentage of outpatient visits for influenza-like illness	1%–3%	4%–6%	7%–9%	10%–12%	≥13%	—	—
**Clinical severity (scale of 1–7)**							
Case-fatality ratio	<0.02%	0.02%–0.05%	0.05%–0.1%	0.1%–0.25%	0.25%–0.5%	0.5%–1%	>1%
Case-hospitalization ratio	<0.5%	0.5%–0.8%	0.8%–1.5%	1.5%–3%	3%–5%	5%–7%	>7%
Deaths-hospitalizations ratio	≤3%	4%–6%	7%–9%	10%–12%	13%–15%	16%–18%	>18%

**Source:** Reed C, Biggerstaff M, Finelli L, et al. Novel framework for assessing epidemiologic effects of influenza epidemics and pandemics. Emerg Infect Dis 2013;19:85–91.

## Methods

### Guidelines Development Process

This 2017 update consists of three separate documents: this report and two supplementary documents (https://stacks.cdc.gov/view/cdc/44313 and https://stacks.cdc.gov/view/cdc/44314). This report provides a brief introduction to pandemic influenza and NPIs; describes the 2007 strategy and the purpose of the updates, particularly after the 2009 H1N1 pandemic; outlines the methods used to develop this update and describe the evidence considered for NPI use during an influenza pandemic; presents CDC’s NPI recommendations; and discusses key areas for further NPI research. The two supplementary documents contain more specific and detailed information about pandemic influenza and NPIs. One document (Technical Report 1 https://stacks.cdc.gov/view/cdc/44313) is divided into chapters and provides an introduction to and overview of NPIs, a description of the new tools developed for pandemic influenza planning and assessment, and a toolbox describing the NPI evidence base, implementation issues, and research gaps. The second document (Technical Report 2 https://stacks.cdc.gov/view/cdc/44314) consists of several appendices that provide a glossary of terms, a detailed description of the methods used for developing the NPI recommendations, a comprehensive summary table of the NPI body of evidence, and a list of tools and resources for pandemic influenza planning and preparedness.

This 2017 update was developed through collaboration involving input from several sources, including peer-reviewed scientific literature, current research, CDC subject-matter experts, and external stakeholders (e.g., federal agencies, public health officials, and business and education partners). Development of these updated guidelines involved participation by multiple CDC groups (e.g., the Community Mitigation Guidelines Work Group and the coordination, abstraction, and consultation teams), as well as a group of external stakeholders who reviewed a document, summarizing the overall direction and key principles and concepts of the guidelines. Input from the work group members, subject-matter experts, and stakeholders was considered and incorporated during the creation of the 2017 planning guidelines. The guidelines were developed during October 2011–October 2016 (Table 7). The complete list of contributors and their roles in the process are available (supplementary Appendix 2 https://stacks.cdc.gov/view/cdc/44314).

**TABLE 7:** Process for developing the community mitigation guidelines for pandemic influenza, October 2011–October 2016

Topic	Comment
Goal of the guidelines	The goal of the 2017 guidelines is to update the 2007 guidance and provide updated recommendations on the use of NPIs during an influenza pandemic in the United States, based on lessons learned from the 2009 H1N1 pandemic and on an expanded evidence base for NPIs that includes studies conducted since 2007.
Users of the guidelines	State, tribal, local, and territorial public health authorities
Population and settings	The updated 2017 planning guidelines apply but are not limited to activities conducted by public health authorities who are responsible for facilitating and implementing emergency preparedness, planning, and response efforts in community settings (e.g., schools, workplaces, and mass gatherings).
Developer of the guidelines	The CDC Community Mitigation Guidelines Work Group convened in October 2012. The group is composed of staff from CDC’s Office of Infectious Diseases, Influenza Coordination Unit, National Center for Emerging and Zoonotic Infectious Diseases, and National Center for Immunization and Respiratory Diseases. The work group members are subject-matter experts in seasonal and pandemic influenza, community mitigation measures, NPIs, epidemiology, health policy, and technical guidelines development. The work group provided technical oversight, coordinated the guidelines development process, and contributed to the writing of the updated guidelines.
Development of the guidelines	The updated planning guidelines are based on a NPI report developed beginning in October 2011 and finalized in August 2013. The NPI report was developed for internal CDC discussions and served as the foundation for updating the NPI recommendations from the 2007 guidance.
Evidence collection	The NPI recommendations in the 2017 guidelines are based on studies published in English-language, peer-reviewed journals through September 2016. The evidence base for NPIs includes systematic literature reviews, metaanalyses, and evidence from epidemiologic studies, laboratory experiments, and modeling simulations.
Method for data synthesis	Staff members from CDC’s Community Interventions for Infection Control Unit worked in pairs to ensure quality control. They reviewed, abstracted, synthesized, and entered approximately 191 articles into spreadsheets to help establish the overall NPI body of literature, including the evidence base for NPIs.
Development of the recommendations	The approach used by the *Guide to Community Preventive Services* (*The Community Guide*) was adapted and applied to develop the NPI recommendations in the updated planning guidelines.
Planning guides	To help operationalize the updated guidelines, six community mitigation prepandemic planning guides have been developed for key populations and decision-makers in community settings. During September–October 2015, before submission for CDC clearance, the National Public Health Information Coalition facilitated discussion of the planning guides by representatives of the public health, education, and business communities. The guides are part of a set of practical, user-friendly, and plain-language companion implementation materials.
Updating the guidelines	The 2017 guidelines will be updated when new information warrants their modification.

**Abbreviation:** NPI = nonpharmaceutical intervention.

### Use of NPIs During Influenza Pandemics

Ten years ago, when the 2007 strategy was being developed, the evidence for the use of NPIs during influenza pandemics was limited, consisting primarily of historical analyses and contemporary observations rather than controlled scientific studies (*45*,*46*). These analyses and observations were supplemented by modeling studies that used historical data to evaluate NPI use in U.S. cities during the 1918 pandemic (*47*,*48*) or that simulated pandemic scenarios as they might occur in the future (*49*–*51*). The simulations, like the historical analyses, generally supported the effectiveness of early, targeted, and phased-in (layered) use of multiple NPIs^§^ in preventing spread of disease, especially when used in combination with antiviral medications (*46*,*49*). This conclusion seemed plausible, confirming the presumption that individual, partially effective NPIs act in complementary ways to decrease various factors that facilitate the spread of influenza under different circumstances and settings (*52*). However, the NPI modeling studies had substantial limitations, including lack of data supporting assumptions about the effectiveness of individual NPIs, economic and social costs of NPIs, and likely rates of compliance (*46*,*49*,*53*).

In 2016, the evidence supporting the effectiveness of NPIs, both when used alone and in combination, was more substantial and included controlled studies evaluating different NPIs. New modeling studies based on data collected during the 2009 H1N1 pandemic response also became available. This update is based on approximately 191 journal articles written in English and published from 1990 through September 2016 that focused on personal protective measures in general; school closure effectiveness and unintended consequences; school absenteeism; spread of disease in child care facilities, colleges, and universities; impact of mass gatherings; and role and impact of NPIs in non–health care workplace settings. These articles were reviewed, abstracted, and synthesized. To assess the strength of the evidence, a five-step NPI rating scheme process was developed by adapting and applying the approach of the *Guide to Community Preventive Services* (*The Community Guide*) (https://www.thecommunityguide.org). Additional information about the NPI rating scheme process is available (supplementary Appendices 3 and 4 https://stacks.cdc.gov/view/cdc/44314).

The selected articles were organized into three groups: 1) personal NPIs (personal protective measures for everyday use and personal protective measures reserved for influenza pandemics); 2) community NPIs (social distancing measures and school closures and dismissals); and 3) environmental NPIs (surface cleaning measures) (Table 8). Key steps included selecting the relevant literature, abstracting and synthesizing the evidence, and assessing the evidence quality (both individual study quality and quality of the body of evidence). A recommendation was formulated based on the evidence of effectiveness for each NPI. The strength of NPI recommendations took into consideration the effectiveness of the intervention, the ease of implementation (including unwanted consequences), and the importance of the intervention as a public health strategy. Additional information about the NPI evidence base is available (supplementary Chapter 3 https://stacks.cdc.gov/view/cdc/44313 and supplementary Appendix 5 https://stacks.cdc.gov/view/cdc/44314).

**TABLE 8:** Number of selected peer-reviewed articles on nonpharmaceutical interventions used to develop community mitigation guidelines for pandemic influenza, by NPI type and measure and by article topic

NPI type and measure	Number and type of articles reviewed*		
	Background	Evidence based^†^	Implementation issues
**Personal NPIs**			
Personal protective measures for everyday use			
Voluntary home isolation^§^	2	7	1
Respiratory etiquette	2	0	3
Hand hygiene	3	15	11
Personal protective measures reserved for pandemics			
Voluntary home quarantine^§^	1	0	3
Use of face masks in community settings	0	18	4
**Community NPIs**			
School closures and dismissals	24	25	26
Social distancing measures for schools, workplaces, and mass gatherings	10	12	11
**Environmental NPIs**			
Environmental surface cleaning measures	1	12	0

**Abbreviation:** NPI = nonpharmaceutical intervention.

*Articles that are cited in more than one section in the supplementary document (supplementary Chapter 3 https://stacks.cdc.gov/view/cdc/44313) are not counted twice in this table.

^†^Of the 89 evidence-based articles, 27 articles assessed the effectiveness of NPIs when used in combination with one another. The evidence-based articles include 14 systematic literature reviews and metaanalyses composed of approximately 475 individual studies that were reviewed and analyzed by their respective authors. These studies contribute to the overall body of literature on NPIs and help support the evidence base on the effectiveness of NPIs. They are provided (supplementary Appendix 6 https://stacks.cdc.gov/view/cdc/44314) but are not accounted for in this table because CDC staff members did not re-review them.

^§^Voluntary home isolation and voluntary home quarantine share the same set of evidence-based articles.

## Recommendations on the Use of Personal, Community, and Environmental NPIs

**FIGURE 5:**
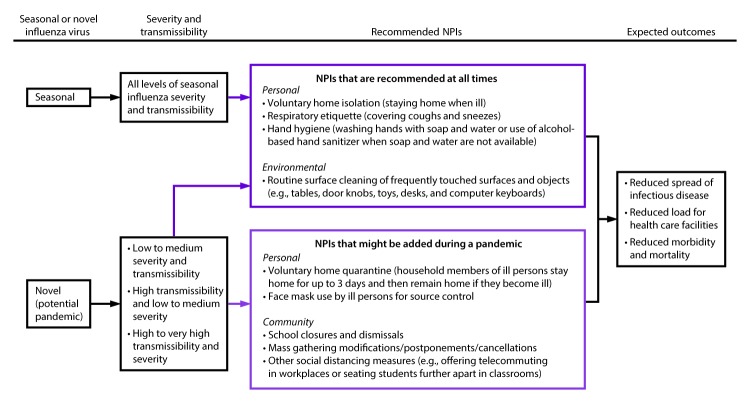
Phased addition of nonpharmaceutical interventions to prevent the spread of pandemic influenza in communities **Abbreviation:** NPI = nonpharmaceutical intervention.

NPIs routinely recommended for prevention of respiratory virus transmission, such as seasonal influenza, include personal protective measures for everyday use (i.e., voluntary home isolation of ill persons, respiratory etiquette, and hand hygiene) and environmental surface cleaning measures (i.e., routine cleaning of frequently touched surfaces and objects). During an influenza pandemic, these NPIs are recommended regardless of the pandemic severity level. Additional personal and community NPIs also might be recommended. Personal protective measures reserved for pandemics include voluntary home quarantine of exposed household members and use of face masks in community settings when ill. Community NPIs might include temporary closures or dismissals of child care facilities and schools with students in grades kindergarten through 12 (K–12), as well as other social distancing measures that increase the physical space between people (e.g., workplace measures such as replacing in-person meetings with teleconferences or modifying, postponing, or cancelling mass gatherings) (Figure 5) (Table 1). Local decisions about NPI selection and timing involve consideration of overall pandemic severity and local conditions (*1*) and require flexibility and possible modifications as the pandemic progresses and new information becomes available.

Updated recommendations on the use of NPIs to help slow the spread and decrease the impact of an influenza pandemic are provided, as is information on the rationale for using each NPI as part of a comprehensive public health strategy for pandemic response and the appropriate settings and use for each NPI according to the severity of the pandemic (Table 9).^¶^ The recommendations that follow are considered an update to the existing recommendations in the 2007 guidance because the same set of NPIs has been maintained and recommended for use early in a pandemic. However, the difference between the guidance issued in 2007 and in 2017 is the clear delineation of NPIs into two categories: 1) NPIs recommended at all times and 2) NPIs recommended for use only during pandemics (based on the level of pandemic severity and local conditions). The 2017 update also provides additional evidence to support the NPI recommendations.

**TABLE 9:** Prepandemic influenza planning scenarios to guide implementation of nonpharmaceutical interventions, by severity of pandemic and the Pandemic Severity Assessment Framework quadrant

Severity of pandemic and PSAF quadrant	Implications of clinical severity and transmissibility in this scenario*	Possible no. of hospitalizations and deaths if unmitigated^,†^ by age group			Historical experience
		Age groups (yrs)	No. of hospitalizations	No. of deaths	
Low to moderate severity (mild to moderate pandemic)	Clinical severity and transmissibility similar to the range seen during annual influenza seasons. Estimated overall attack and case-fatality rates: 18% and 0.03%, respectively. Rates of severe outcomes are greater among younger persons than during influenza seasons.	**All ages**	**340,000**	**17,000**	**2009 pandemic** • First detected in North America, the 2009 H1N1 pandemic quickly spread to all continents. In the United States, persons at high risk for severe complications included pregnant women and those with neuromuscular disease, lung disease, morbid obesity, and other chronic conditions. An estimated 43–89 million people in the United States became ill with H1N1 from April 2009 through April 2010, and approximately 12,000 people died.^§^ A total of 87% of deaths were among persons aged ≤65 yrs, with a mean age of 43 yrs.^¶^ During typical influenza seasons, 80%–90% of deaths are among persons aged ≥65 yrs, and the mean age of influenza-related deaths is approximately 76 yrs.**
		0–18	50,000	1,000	
PSAF quadrant: A		18–64	135,000	6,000	
		≥65	155,000	10,000	
Moderate to high severity (moderate to severe pandemic)	Clinical severity similar to the range seen during annual influenza seasons. Transmissibility greater than during influenza seasons. Estimated overall attack and case–fatality rates: 22% and 0.05%, respectively. Rates of severe outcomes are greater than during influenza seasons, especially among younger persons.	**All ages**	**550,000**	**35,000**	**1968 pandemic** • First detected in Hong Kong in July 1968, a new influenza virus (H3N2) spread worldwide. The first cases in the United States were detected in September 1968. The 1968 influenza pandemic resulted in approximately 30,000 deaths in the United States, with approximately half among those aged ≥65 yrs.^††^^,^^§§^
		0–18	80,000	2,500	
PSAF quadrant: B		18–64	220,000	12,000	
		≥65	250,000	20,000	
High severity (severe pandemic)	Clinical severity similar to the range seen during annual influenza seasons. Transmissibility greater than during influenza seasons. Estimated overall attack and case-fatality rates: 28% and 0.1%, respectively. Rates of severe outcomes are greater than during influenza seasons.	**All ages**	**1,100,000**	**86,000**	**1957 pandemic** • A new influenza virus, H2N2 (the Asian strain), emerged in China in February 1957 and spread to approximately 20 countries, including the United States, by June 1957. An estimated 25% of the U.S. population became ill with the new pandemic virus strain. U.S. infection rates were highest among school-aged children and adults aged ≤40 yrs, with most (64%) of the approximately 70,000 deaths occurring among older adults.^††^^,^^§§^^,^^¶¶^
		0–18	150,000	6,000	
PSAF quadrant: B		18–64	450,000	30,000	
		≥65	500,000	50,000	
Very high severity (very severe to extreme pandemic)	Both clinical severity and transmissibility are greater than during annual influenza seasons. Estimated overall attack and case-fatality rates: 30% and 1.5%, respectively. Rates of severe outcomes are greater than during influenza seasons, especially among young adults.	**All ages**	**7,500,000**	**1,400,000**	**1918 pandemic** • The 1918 pandemic resulted in death for 2%–3% of those infected, a case-fatality rate that was much greater than the rate during an average influenza season. The pandemic virus was easily transmitted. Approximately one fourth of the U.S. population became ill, and approximately 500,000 died; 99% of deaths occurred in persons aged ≤65 yrs.^††^^,^***
		0–18	1,000,000	100,000	
PSAF quadrant: D		18–64	3,000,000	500,000	
		≥65	3,400,000	800,000	

**Abbreviation:** PSAF = Pandemic Severity Assessment Framework.

*Based on PSAF (**Source:** Reed C, Biggerstaff M, Finelli L, et al. Novel framework for assessing epidemiologic effects of influenza epidemics and pandemics. Emerg Infect Dis 2013;19:85–91). Age-specific point estimates of hospitalizations and deaths are based on U.S. Census 2010 population data.

^†^Point estimates for hospitalizations and deaths, by age group, are based on the estimated overall attack and case-fatality rates provided in the second column (clinical severity and transmissibility). Age-specific point estimates of hospitalizations and deaths are based on U.S. Census 2010 population data.

^§^**Source:** Shrestha SS, Swerdlow DL, Borse RH, et al. Estimating the burden of 2009 pandemic influenza A (H1N1) in the United States (April 2009–April 2010). Clin Infect Dis 2011;52(Suppl 1):S75–S82.

^¶^**Source:** Fowlkes AL, Arguin P, Biggerstaff MS, et al. Epidemiology of 2009 pandemic influenza A (H1N1) deaths in the United States, April–July 2009. Clin Infect Dis 2011;52(Suppl 1):S60–S68.

**Source: Viboud C, Miller M, Olson DR, Osterholm M, Simonsen L. Preliminary estimates of mortality and years of life lost associated with the 2009 A/H1N1 pandemic in the U.S. and comparison with past influenza seasons. PLoS Currents 2010;2:RRN1153.

^††^**Source:** Simonsen L, Clarke MJ, Schonberger LB, Arden NH, Cox NJ, Fukuda K. Pandemic versus epidemic influenza mortality: a pattern of changing age distribution. J Infect Dis 1998;178:53–60.

^§§^**Source:** Cox NJ, Subbarao K. Global epidemiology of influenza: past and present. Annu Rev Med 2000;51:407–21.

^¶¶^**Source:** Henderson DA, Courtney B, Inglesby TV, Toner E, Nuzzo JB. Public health and medical responses to the 1957–58 influenza pandemic. Biosecur Bioterror 2009;7:265–73.

***Source: Collins SD. Age and sex incidence of influenza and pneumonia morbidity and mortality in the epidemic of 1928–29 with comparative data for the epidemic of 1918–19. Public Health Rep 1931;46:1909–37.

### Personal NPIs

NPIs that can be implemented by individual persons include the following:

- **Personal protective measures for everyday use:** These include voluntary home isolation of ill persons, respiratory etiquette, and hand hygiene.
- **Personal protective measures reserved for pandemics:** These include voluntary home quarantine of exposed household members and use of face masks in community settings when ill.

#### Personal Protective Measures for Everyday Use

Personal protective measures are preventive actions that can be used daily to slow the spread of respiratory viruses (https://www.cdc.gov/nonpharmaceutical-interventions/personal/index.html; supplementary Chapter 3 https://stacks.cdc.gov/view/cdc/44313). These measures include the following:

- **Voluntary home isolation (i.e., staying home when ill or self-isolation):** Persons with influenza stay home for at least 24 hours after a fever or signs of a fever (chills, sweating, and feeling warm or flushed)** are gone (https://www.cdc.gov/flu/protect/preventing.htm), except to obtain medical care or other necessities.^††^ To ensure that the fever is gone, patients’ temperature should be measured in the absence of medication that lowers fever (e.g., acetaminophen or ibuprofen). In addition to fever, common influenza symptoms include cough or chest discomfort, muscle or body aches, headache, and fatigue. Persons also might experience sneezing, a runny or stuffy nose, sore throat, vomiting, and diarrhea (https://www.cdc.gov/flu/consumer/symptoms.htm).
- **Respiratory etiquette:** Persons cover coughs and sneezes, preferably with a tissue, and then dispose of tissues and disinfect hands immediately after a cough or sneeze, or (if a tissue is not available) cough or sneeze into a shirt sleeve. Touching the eyes, nose, and mouth should be avoided to help slow the spread of germs (https://www.cdc.gov/flu/protect/covercough.htm).
- **Hand hygiene:** Persons perform regular and thorough hand washing with soap and water (or use alcohol-based hand sanitizers containing at least 60% ethanol or isopropanol when soap and water are not available).

**Rationale for use as a public health strategy**. Most persons infected with an influenza virus might become infectious 1 day before the onset of symptoms and remain infectious up to 5–7 days after becoming ill (*54*,*55*). However, studies found that infants and immunocompromised persons might shed influenza viruses for prolonged periods (up to 21 days and a mean of 19 days, respectively) (*56*,*57*). The effectiveness of personal protective measures depends on their ability to interrupt virus transmission from one person to another. Voluntary home isolation, which is a form of patient isolation, prevents an ill person from infecting other people outside of their household.^§§^ Respiratory etiquette reduces the dispersion of droplets contaminated with influenza virus being propelled through the air by coughing or sneezing. Hand hygiene reduces the transmission of influenza viruses that occurs when one person touches another (e.g., with a contaminated hand). Contamination also can occur through self-inoculation via fomite transmission (indirect contact transmission) when persons touch a contaminated surface and then touch their nose with a contaminated hand. A study conducted in households in Bangkok, Thailand, found that increased handwashing reduced surface contamination with influenza virus, which lowered the potential for self-inoculation via fomite transmission (*58*). Additional studies found that influenza viruses can remain viable on the human hand for roughly 3–5 minutes (*59*) and that influenza viruses can remain on fingers for 30 minutes after contamination (*60*).

**Settings and use**. Voluntary home isolation involves persons remaining at home when ill with influenza. Respiratory etiquette and hand hygiene are recommended in homes and in all other community settings, including schools and workplaces. All three personal protective measures are considered everyday preventive actions that should be implemented year-round but that are especially important during annual influenza seasons and influenza pandemics (Table 10). Use of these personal protective measures might result in some secondary (unintended or unwanted) consequences (e.g., concerns about job security for ill persons who lack paid sick leave or skin irritations due to frequent hand washing).

**TABLE 10:** Recommended nonpharmaceutical interventions for influenza pandemics, by setting and pandemic severity*

Setting	Pandemic severity		
	Low to moderate severity (mild to moderate pandemic)	High severity (severe pandemic)	Very high severity (very severe to extreme pandemic^†^)
All	CDC recommends voluntary home isolation of ill persons, respiratory etiquette, hand hygiene, and routine cleaning of frequently touched surfaces and objects.**^§^**	CDC recommends voluntary home isolation of ill persons, respiratory etiquette, hand hygiene, and routine cleaning of frequently touched surfaces and objects.	CDC recommends voluntary home isolation of ill persons, respiratory etiquette, hand hygiene, and routine cleaning of frequently touched surfaces and objects.
Residences	CDC generally does not recommend voluntary home quarantine of exposed household members.	CDC might recommend voluntary home quarantine of exposed household members in areas where novel influenza virus circulates.	CDC might recommend voluntary home quarantine of exposed household members in areas where novel influenza virus circulates.
	CDC generally does not recommend use of face masks by ill persons.	CDC might recommend use of face masks by ill persons when crowded community settings cannot be avoided.	CDC might recommend use of face masks by ill persons when crowded community settings cannot be avoided.
Child care facilities, schools for grades K–12, and colleges and universities	CDC might recommend selective school dismissals in facilities serving children at high risk for severe influenza complications.	CDC might recommend temporary preemptive, coordinated dismissals of child care facilities and schools.^¶^	CDC might recommend temporary preemptive, coordinated dismissals of child care facilities and schools.
		If schools remain open, CDC might recommend social distancing measures.**	If schools remain open, CDC might recommend social distancing measures.
Workplaces	CDC generally does not recommend social distancing measures.	CDC might recommend social distancing measures.^††^	CDC might recommend social distancing measures.
Mass gatherings^§§^	CDC generally does not recommend modifications, postponements, or cancellations.	CDC might recommend modifications, postponements, or cancellations.	CDC might recommend modifications, postponements, or cancellations.

**Abbreviation:** NPI = nonpharmaceutical intervention.

*Personal, community, and environmental NPIs should be 1) initiated early in a pandemic before local epidemics begin to grow exponentially, 2) targeted toward the nexus of transmission (in affected areas where the novel virus circulates), and 3) layered together to reduce community transmission to the greatest extent possible.

^†^During a very severe or extreme pandemic (similar to the 1918 pandemic), CDC is likely to take an aggressive stance and recommend certain additional NPIs.

**^§^**Recommended NPIs are the same for seasonal influenza.

^¶^Preemptive, coordinated dismissals might be implemented early during a pandemic to decrease the spread of influenza before many students and staff members become ill. Selective dismissals might be implemented by schools that serve students at high risk for complications from infection with influenza. Reactive dismissals might be implemented when many students and staff members are ill and not attending school or when many students and staff members are arriving at school ill and being sent home. Selective and reactive dismissals do not help slow disease transmission in the community.

**Social distancing measures that reduce face-to-face contact in schools might include dividing classes into smaller groups of students who are spaced further apart from each other within the classroom.

^††^Social distancing measures that reduce face-to-face contact in workplaces might include offering telework and remote meeting options. Flexible sick leave policies should be implemented to encourage workers to stay home if needed.

^§§^In all scenarios, mass gathering attendance during local outbreaks should be discouraged for persons at high risk for severe influenza-related complications.

**CDC recommendations**

**Voluntary home isolation:** CDC recommends voluntary home isolation of ill persons (staying home when ill) year-round and especially during annual influenza seasons and influenza pandemics.

**Respiratory etiquette and hand hygiene:** CDC recommends respiratory etiquette and hand hygiene in all community settings, including homes, child care facilities, schools, workplaces, and other places where people gather, year-round and especially during annual influenza seasons and influenza pandemics.

#### Personal Protective Measures Reserved for Pandemics

Voluntary home isolation, respiratory etiquette, and hand hygiene are recommended during both annual influenza seasons and influenza pandemics. Additional personal protective measures that might be recommended during pandemics include voluntary home quarantine of exposed household members and the use of face masks in community settings when ill. These measures might contribute to reductions in transmission of pandemic influenza viruses when the level of pandemic severity and local conditions warrant their use (supplementary Chapter 3 https://stacks.cdc.gov/view/cdc/44313).

##### Voluntary Home Quarantine

Voluntary home quarantine of non-ill household members of persons with influenza (also called self-quarantine or household quarantine) helps prevent disease spread from households to schools, workplaces, and other households because those household members have been exposed to the influenza virus. Exposed household members of symptomatic persons (with confirmed or probable pandemic influenza) should stay home for up to 3 days (the estimated incubation period for seasonal influenza) (*61*) starting from their initial contact with the ill person. If they then become ill, they should practice voluntary home isolation (i.e., they should remain at home until recovered as discussed previously; https://www.cdc.gov/quarantine/index.html). For certain exposed household members (e.g., those at high risk for influenza complications or with severe immune deficiencies), guidelines should be consulted regarding the prophylactic use of antiviral medications (https://www.cdc.gov/flu/professionals/antivirals/index.htm).

**Rationale for use as a public health strategy**. Voluntary home quarantine might help slow a pandemic by reducing community transmission from households with a person who has influenza because the exposed household members are at increased risk for infection. Furthermore, certain infected (but not yet symptomatic) household members could begin shedding influenza virus at least a day before exhibiting symptoms and could infect friends, neighbors, and others in the community (e.g., at school or work) before becoming symptomatic. Therefore, all members of a household with a symptomatic person (with confirmed or probable pandemic influenza) might be asked to stay home for a specified period of time (up to 3 days) to assess for early signs and symptoms of pandemic influenza virus infection. If other household members become ill during this period, then the time for voluntary home quarantine might need to be extended for another incubation period. The evidence for voluntary home quarantine, particularly when used in combination with other NPIs, includes a systematic literature review, historical analyses of the 1918 pandemic, and mathematical modeling studies (supplementary Chapter 3 https://stacks.cdc.gov/view/cdc/44313 and supplementary Appendix 5 https://stacks.cdc.gov/view/cdc/44314).

**Settings and use.** Voluntary home quarantine of exposed household members might be recommended during severe, very severe, or extreme influenza pandemics (Table 10) to help reduce the chance of transmitting the virus to others outside of the household. Advance planning is needed to minimize potential secondary consequences for persons who have special cultural, economic, legal, mental, physical, or social status needs (e.g., older adults who depend on necessary community-based services such as home-delivered meals and transportation to health care services). Other secondary consequences might include missed work and loss of income for persons whose employers do not have paid sick leave policies that include home quarantine during pandemics.

**CDC recommendations**

**Voluntary home quarantine:** CDC might recommend voluntary home quarantine of exposed household members as a personal protective measure during severe, very severe, or extreme influenza pandemics in combination with other personal protective measures such as respiratory etiquette and hand hygiene. If a member of the household is symptomatic with confirmed or probable pandemic influenza, then all members of the household should stay home for up to 3 days (the estimated incubation period for seasonal influenza)^,¶^^¶^ starting from their initial contact with the ill person, to monitor for influenza symptoms.

##### Use of Face Masks in Community Settings

Face masks (disposable surgical, medical, or dental procedure masks) are widely used by health care workers to prevent respiratory infections both in health care workers and patients. They also might be worn by ill persons during severe, very severe, or extreme pandemics to prevent spread of influenza to household members and others in the community. However, little evidence supports the use of face masks by well persons in community settings, although some trials conducted during the 2009 H1N1 pandemic found that early combined use of face masks and other NPIs (such as hand hygiene) might be effective (supplementary Chapter 3 https://stacks.cdc.gov/view/cdc/44313).

**Rationale for use as a public health strategy.** Face masks provide a physical barrier that prevents the transmission of influenza viruses from an ill person to a well person by blocking large-particle respiratory droplets propelled by coughing or sneezing. Face mask use by well persons is not routinely needed in most situations to prevent acquiring the influenza virus. However, use of face masks by well persons might be beneficial in certain situations (e.g., when persons at high risk for influenza complications cannot avoid crowded settings or parents are caring for ill children at home). Face mask use by well persons also might reduce self-inoculation (e.g., touching the nose with the hand after touching a contaminated surface).

**Settings and use.** Disposable surgical, medical, and dental procedure masks are used widely in health care settings to prevent exposure to respiratory infections. Face masks have few secondary consequences (e.g., discomfort or difficulty breathing) when worn properly and consistently, and face masks sized for children are available. (Additional information about face masks is available at https://www.fda.gov/medicaldevices/productsandmedicalprocedures/generalhospitaldevicesandsupplies/personalprotectiveequipment/ucm055977.htm and https://www.osha.gov/Publications/respirators-vs-surgicalmasks-factsheet.html.)

**CDC recommendations**

**Use of face masks by ill persons:** CDC might recommend the use of face masks by ill persons as a source control measure during severe, very severe, or extreme influenza pandemics when crowded community settings cannot be avoided (e.g., when adults and children with influenza symptoms seek medical attention) or when ill persons are in close contact with others (e.g., when symptomatic persons share common spaces with other household members or symptomatic postpartum women care for and nurse their infants). Some evidence indicates that face mask use by ill persons might protect others from infection.

**Use of face masks by well persons:** CDC does not routinely recommend the use of face masks by well persons in the home or other community settings as a means of avoiding infection during influenza pandemics except under special, high-risk circumstances (https://www.cdc.gov/flu/professionals/infectioncontrol/maskguidance.htm). For example, during a severe pandemic, pregnant women and other persons at high risk for influenza complications might use face masks if unable to avoid crowded settings, especially if no pandemic vaccine is available. In addition, persons caring for ill family members at home (e.g., a parent of a child exhibiting influenza symptoms) might use face masks to avoid infection when in close contact with a patient, just as health care personnel wear masks in health care settings.

### Community NPIs

NPIs that can be implemented by communities include the following:

- **School closures and dismissals:** These include temporary closures and dismissals of child care facilities, K–12 schools, and institutions of higher education.
- **Social distancing measures:** These include measures for schools, workplaces, and mass gatherings.

#### School Closures and Dismissals

In the event of a pandemic, state and local public health authorities play an important role in protecting the school community and should establish and maintain partnerships with district and school leaders, school emergency operations planning teams, and local municipality leaders (e.g., mayors). Public health authorities are a credible source of information, have multiple (often free) resources available for information awareness campaigns, and provide guidance for increasing school response measures. Depending on the severity of the pandemic, these measures might range from everyday preventive actions to preemptive, coordinated school closures and dismissals. A school closure means closing a school and sending all the students and staff members home, whereas during a school dismissal, a school might stay open for staff members while the children stay home. Preemptive school dismissals can be used to disrupt transmission of influenza before many students and staff members become ill. Coordinated dismissals refer to the simultaneous or sequential closing of schools in a jurisdiction. Thus, preemptive, coordinated school closures and dismissals can be used early during an influenza pandemic to prevent virus transmission in schools and surrounding communities by reducing close contact among the following groups (supplementary Chapter 3 https://stacks.cdc.gov/view/cdc/44313):

- Children in child care centers and preschools
- School-aged children and teens in K–12 schools
- Young adults in institutions of higher education

During a dismissal, the school facilities are kept open, which allows teachers to develop and deliver lessons and materials, thus maintaining continuity of teaching and learning, and allows other staff members to continue to provide services and help with additional response efforts. School closures and dismissals might be coupled with social distancing measures (e.g., cancelling sporting events and other mass gatherings) to reduce out-of-school social contact among children when schools are closed.

**Rationale for use as a public health strategy.** Preventing the spread of disease in educational settings among children and young adults reduces the risk for infection for these age groups and slows virus transmission in the community. Components of the strategy might include preemptive, coordinated school closures and dismissals implemented during the earliest stages of a pandemic, before many students and staff members become ill. Preemptive, coordinated dismissals can be implemented by the following facilities for the following reasons:

- Child care facilities and K–12 schools
  - Children have higher influenza attack rates than adults (*62*) and are infectious for a longer period than adults (*63*,*64*).
  - Influenza transmission is common in schools and contributes to school absenteeism and parental absenteeism from work (*65*,*66*).
  - The presence of school-aged children in a household is a risk factor for influenza virus infection in families (*62*,*65*,*67*).
  - Social contact and mixing patterns among school-aged children differ substantially depending on the grade and school level, during various periods of the school day, between weekdays and weekends, and between regular school terms and holiday breaks (*68*–*71*). Physical floor plans and intergrade activities (e.g., cafeteria size and lunch breaks) also can affect in-school social mixing (*68*).
  - Schoolchildren can introduce the influenza virus into a community, leading to increased rates of illness among their household or community contacts (*72*–*74*).
- Institutions of higher education
  - Influenza outbreaks on college and university campuses typically have high attack rates (44%–73%) (*75*–*78*) and cause substantial morbidity (*79*,*80*). For example, during the 2009 H1N1 pandemic, influenza spread rapidly through a university campus within 2 weeks (*81*); on another residential campus, one infected freshman initiated an outbreak that resulted in 226 laboratory-confirmed cases. Freshmen were the main facilitators of the spread of the H1N1pdm09 virus because of their higher number and frequency of social contacts (*82*).
  - Influenza is more prevalent among residential students at boarding schools and colleges than among nonresidential students (*78*,*83*).
  - ILIs are common among college and university students and are associated with increased health care use, decreased health status, and impaired school performance (*84*).

Implementation of preemptive, coordinated school closures and dismissals during an evolving influenza pandemic might have one or more of the following three public health objectives***:

- **Objective 1:** To gain time for an initial assessment of transmissibility and clinical severity of the pandemic virus in the very early stage of its circulation in humans (closures for up to 2 weeks)
- **Objective 2:** To slow down the spread of the pandemic virus in areas that are beginning to experience local outbreaks and thereby allow time for the local health care system to prepare additional resources for responding to increased demand for health care services (closures up to 6 weeks)
- **Objective 3:** To allow time for pandemic vaccine production and distribution (closures up to 6 months)

Two other types of school closures and dismissals might be implemented during a pandemic for public health or institutional reasons. These interventions do not slow disease spread in the community; therefore, they are not considered NPIs. They include the following:

- **Selective school closures and dismissals:** These might be implemented by schools that serve students at high risk for complications from infection with influenza^,†^^††^ especially when transmission rates are high. For example, a school that serves children with certain medical conditions or pregnant teens might decide to close while other schools in the area remain open. In addition, some communities or early childhood programs might consider closing child care facilities to help decrease the spread of influenza among children aged <5 years. Selective dismissals are intended to protect persons at high risk for influenza rather than to help reduce virus transmission within the community.
- **Reactive school closures and dismissals:** These might be implemented when many students and staff members are ill and not attending school or when many students and staff members are arriving at school ill and being sent home. For example, a child care center might close because it is unable to operate under these conditions. Reactive dismissals, which might occur during outbreaks of seasonal influenza (*85*) and during pandemics (*15*), are unlikely to affect virus transmission because they typically take place after considerable, if not widespread, transmission has already occurred in the community. For example, a 4-day reactive closure in a western Kentucky school district did not reduce ILI transmission in the rural community (*86*). Similarly, closing 559 Michigan schools at least once during the fall wave (i.e., second wave) of the 2009 H1N1 pandemic had little effect on community levels of ILI (*87*).

For more information about preparing for influenza and the different types of dismissals, see CDC websites regarding 1) child care facilities (https://www.cdc.gov/h1n1flu/childcare/toolkit/pdf/childcare_toolkit.pdf), 2) K–12 schools (https://www.cdc.gov/h1n1flu/schools/toolkit/pdf/schoolflutoolkit.pdf), and 3) institutions of higher education (https://www.cdc.gov/h1n1flu/institutions/toolkit/pdf/IHE_toolkit.pdf).

**Settings and use.** Preemptive, coordinated school closures and dismissals might be implemented at child care facilities, K–12 schools, and institutions of higher education. They are most likely to be implemented when an influenza pandemic is severe, very severe, or extreme (Table 10). Secondary consequences include missed work and loss of income for parents who stay home from work to care for their children and missed opportunities to vaccinate school-aged children rapidly unless other mechanisms are considered.

**CDC recommendations**

**School closures and dismissals:** CDC might recommend the use of preemptive, coordinated school closures and dismissals during severe, very severe, or extreme influenza pandemics. This recommendation is in accord with the conclusions of the U.S. Community Preventive Services Task Force (https://www.thecommunityguide.org/findings/emergency-preparedness-and-response-school-dismissals-reduce-transmission-pandemic-influenza), which makes the following recommendations:

- The task force recommends preemptive, coordinated school dismissals during a severe influenza pandemic.
- The task force found insufficient evidence to recommend for or against preemptive, coordinated school dismissals during a mild or moderate influenza pandemic. In these instances, jurisdictions should make decisions that balance local benefits and potential harms.

#### Social Distancing Measures for Schools, Workplaces, and Mass Gatherings

Social distancing measures can reduce virus transmission by decreasing the frequency and duration of social contact among persons of all ages. These measures are common-sense approaches to limiting face-to-face contact, which reduces person-to-person transmission.

**Rationale for use as a public health strategy.** Social distancing measures that reduce opportunities for person-to-person virus transmission can help delay the spread and slow the exponential growth of a pandemic. The optimal strategy is to implement these measures simultaneously in places where persons gather. Although direct evidence is limited for the effectiveness of these measures, components of the strategy might include reducing social contacts at the following places:

- **Schools:** Children have higher influenza attack rates than adults, and influenza transmission is common in schools.
- **Workplaces:** More than half of all U.S. adults participate in the U.S workforce^,§^^§§^ and workers often share office space and equipment and have frequent face-to-face contact. Influenza attack rates in working-age adults (aged 18–64 years) might be as high as 15.5% during a single influenza season (*88*).
- **Mass gatherings:** Group events such as concerts, festivals, and sporting events bring people into close contact for extended periods (*89*–*92*). A systematic literature review of respiratory disease outbreaks related to mass gatherings in the United States during 2005–2014 indicated that 40 of 72 different outbreaks were associated with state or county agriculture fairs and (zoonotic) transmission of influenza A H3N2v, and 25 outbreaks were associated with residential youth summer camps and person-to-person transmission of influenza A H1N1 (*93*). An infected traveler attending a mass gathering might introduce influenza to a previously unaffected area, and a person who becomes infected at the event can further spread the infection after returning home (*89*,*90*,*92*,*94*–*96*). Even when a circulating virus has a relatively low basic reproductive rate (*R_0_*), intensely crowded settings might lead to high secondary attack rates (*92*). For example, during the 2013 Hajj (Islamic pilgrimage to Mecca) in Saudi Arabia, influenza A/H1N1 virus was found in only two Indonesians on arrival but spread to 25 persons from Africa, Central Asia, and Southeast Asia after the Hajj because of the extremely crowded conditions when performing rituals (*97*).

Multiple social distancing measures can be implemented simultaneously. Although there is limited empirical evidence supporting the effectiveness of implementing any individual measure alone (other than school closures and dismissals), the evidence for implementing multiple social distancing measures in combination with other NPIs includes systematic literature reviews, historical analyses of the 1918 pandemic, and mathematical modeling studies (supplementary Chapter 3 https://stacks.cdc.gov/view/cdc/44313 and supplementary Appendix 5 https://stacks.cdc.gov/view/cdc/44314).

**Settings and use*.*** Social distancing measures can be implemented in a range of community settings, including educational facilities, workplaces, and public places where people gather (e.g., parks, religious institutions, theaters, and sports arenas). The choice of social distancing measure depends on the severity of the pandemic (Table 10). Certain measures might be implemented with few secondary consequences (e.g., increased use of e-mail and teleconferences in some workplaces), whereas others might require advance planning (e.g., modification of mass gatherings). Examples of practical measures that might reduce face-to-face contact in community settings include the following:

- If schools remain open during a pandemic, divide school classes into smaller groups of students and rearrange desks so students are spaced at least 3 feet (*98*) from each other in a classroom.
- Offer telecommuting and replace in-person meetings in the workplace with video or telephone conferences.
- Modify, postpone, or cancel mass gatherings.

**CDC recommendations**

**Social distancing measures:** Even though the evidence base for the effectiveness of some of these measures is limited, CDC might recommend the simultaneous use of multiple social distancing measures to help reduce the spread of influenza in community settings (e.g., schools, workplaces, and mass gatherings) during severe, very severe, or extreme influenza pandemics while minimizing the secondary consequences of the measures. Social distancing measures include the following:

- Increasing the distance to at least 3 feet (*98*) between persons when possible might reduce person-to person transmission. This applies to apparently healthy persons without symptoms. In the event of a very severe or extreme pandemic, this recommended minimal distance between people might be increased.
- Persons in community settings who show symptoms consistent with influenza and who might be infected with (probable) pandemic influenza should be separated from well persons as soon as practical, be sent home, and practice voluntary home isolation.

### Environmental NPIs: Environmental Surface Cleaning Measures

Environmental surface cleaning measures can help eliminate influenza viruses from frequently touched surfaces and objects, including tables, door knobs, toys, desks, and computer keyboards. These measures involve cleaning surfaces with detergent-based cleaners or disinfectants that have been registered with the Environmental Protection Agency*.*^¶¶¶^

**Rationale for use as a public health strategy.** Although the percentage of influenza cases involving contact transmission (i.e., hand transfer of virus from contaminated objects to the eyes, nose, or mouth) is unknown, this mode of transmission is a recognized route of virus spread (*99*). The routine use of cleaning measures that eliminate viruses from contaminated surfaces might reduce the spread of influenza viruses (supplementary Chapter 3 https://stacks.cdc.gov/view/cdc/44313).

**Settings and use.** Environmental surface cleaning measures are recommended for frequently touched surfaces and objects in homes, child care facilities, schools, workplaces, and other places where persons gather. These measures can be used for prevention of seasonal influenza and in all pandemic severity scenarios (Table 10). Use of these measures might result in some secondary consequences (e.g., failing to read instruction labels before applying disinfectants to ensure that they are safe and appropriate to use or cleaning with poor ventilation during the application process).

**CDC recommendations**

**Environmental surface cleaning measures:** CDC recommends environmental surface cleaning measures in all settings, including homes, schools, and workplaces, to remove influenza viruses from frequently touched surfaces and objects. Use of these measures might help prevent transmission of various infectious agents, including seasonal and pandemic influenza (https://www.cdc.gov/nonpharmaceutical-interventions/environmental/index.html; https://www.cdc.gov/oralhealth/infectioncontrol/questions/cleaning-disinfecting-environmental-surfaces.html).

Additional guidance is available from CDC for health care facilities (https://www.cdc.gov/hicpac/pdf/guidelines/eic_in_HCF_03.pdf), schools (https://www.cdc.gov/flu/school/cleaning.htm), and airline, travel, and transportation industries (https://www.cdc.gov/flu/pandemic-resources/archived/transportation-planning.html).

## Discussion

This report expands the NPI guidance presented in the 2007 report by providing evidence-based recommendations on the use of the same set of NPIs. These NPIs include personal protective measures for everyday use and for use during a pandemic, community measures (school closures and dismissals and social distancing), and environmental surface cleaning measures.

### Key Concepts Maintained from 2007 Guidance

The rationale for and key concepts regarding the use of NPIs during influenza pandemics first presented in the 2007 guidance remain unchanged. Because production of a pandemic vaccine can take up to 6 months and antiviral medications might be prioritized for treatment, NPIs are likely to be the only prevention tools available early in a pandemic. Therefore, they are critical to slowing the spread of the pandemic influenza virus while a pandemic vaccine is under development.

Like the 2007 strategy, this 2017 update affirms the importance of prepandemic planning and preparedness for use of NPIs during a pandemic response and recommends the early, targeted, and simultaneous implementation of multiple NPIs to decrease influenza virus transmission. Although community-level NPIs can help slow virus transmission, as supported by historical information (*100*), empirical observations (*101*), and mathematical modeling (*102*), these measures are likely to cause unwanted consequences by introducing new norms for social behavior (e.g., adopting precautionary health-protective behaviors such as limiting face-to-face contact with family and friends, only shopping for essential items, avoiding places where people congregate, or not using public transportation) (*103*), interrupting routine societal functions, and entailing additional costs. If an evolving influenza pandemic is characterized by high clinical severity, the benefits of deploying NPIs, including those with greater potential for secondary consequences, are likely to outweigh potential harms. The more difficult decision is determining how and when to implement the community-level NPIs that are more disruptive to society (e.g., temporary K–12 school closures) during pandemics of moderate severity. In each locality, the goal should be to implement NPIs early enough and long enough to maximize effectiveness while minimizing economic and social costs to ensure that NPIs are commensurate to the pandemic severity.

### New Elements Added in 2017

New elements in this report, in addition to the evidence-based NPI recommendations, include a summary of key lessons learned from the 2009 H1N1 pandemic response (Box 1), information on community engagement and preparedness (supplementary Chapter 1 https://stacks.cdc.gov/view/cdc/44313), and information on new or updated pandemic assessment tools (supplementary Chapter 2 https://stacks.cdc.gov/view/cdc/44313), which include the novel influenza virus pandemic intervals tool, the Influenza Risk Assessment Tool, and PSAF. As described in the following sections, this report also presents two additional planning tools designed to assist states and localities in ensuring pandemic preparedness.

#### Prepandemic Planning Scenarios for NPI Implementation According to Pandemic Severity

**FIGURE 6:**
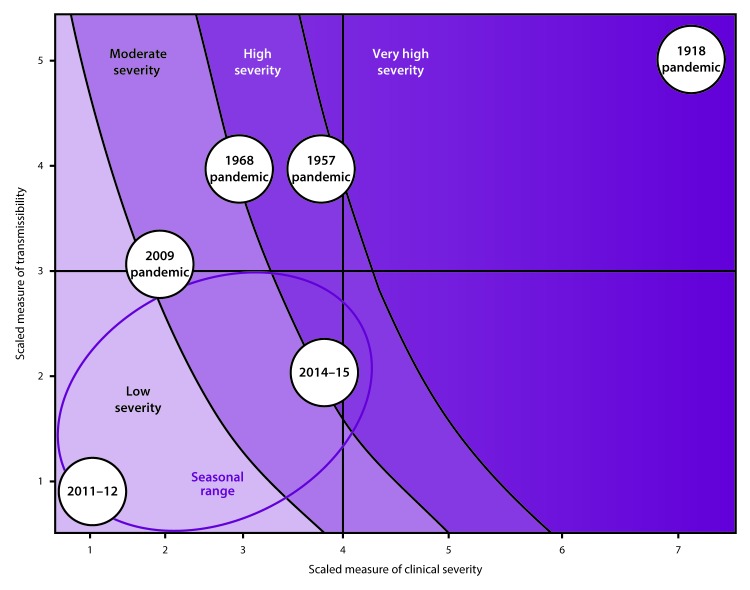
U.S. Department of Health and Human Services pandemic planning scenarios based on the Pandemic Severity Assessment Framework **Source:** Adapted from: Reed C, Biggerstaff M, Finelli L, et al. Novel framework for assessing epidemiologic effects of influenza epidemics and pandemics. Emerg Infect Dis 2013;19:85–91.

During the initial stages of a pandemic, CDC will use the PSAF tool to prepare an initial assessment of pandemic severity that provides early guidance on use of NPIs to help slow the transmission of the novel virus. To facilitate the use of the initial assessment information by state and local health departments, CDC has provided a set of four prepandemic planning scenarios. Each scenario aligns with one of the four assessment quadrants (Figure 3) and provides information on past influenza pandemics for comparison (Table 9). These planning scenarios are designed to facilitate state and local prepandemic planning for NPI implementation according to pandemic severity (as classified by PSAF) (Figure 6) (Tables 9 and 10). After sufficient epidemiologic data are accrued and the refined assessment of pandemic severity becomes available, CDC will issue updated pandemic NPI guidance, which will be tailored more precisely to the specific pandemic. Additional information about the planning scenarios and phasing of NPIs is available (supplementary Chapter 2 https://stacks.cdc.gov/view/cdc/44313).

#### Supplemental Prepandemic NPI Planning Guides

The 2007 report included supplemental prepandemic NPI planning guides for individuals and families; child care programs, K–12 schools, and institutions of higher education; community- and faith-based organizations; and businesses and other workplaces. These guides have been updated, and two new guides have been developed for public health communicators and event planners that address NPI communications and modification, postponement, or cancellation of mass gatherings. These guides are intended to help operationalize the 2017 update and provide specific information that can assist different groups in their prepandemic planning and decision-making (https://www.cdc.gov/nonpharmaceutical-interventions).

### Future Research

Although progress has been made since 2009 toward building the evidence base for use of NPIs to slow the spread of pandemic influenza, additional research is needed. For personal NPIs, areas for additional research include evaluating the effects of increased frequency and quality of hand washing on influenza virus transmission, determining the role of infected persons who are not symptomatic in the transmission of influenza viruses in households, and assessing the effectiveness, acceptability, and feasibility of recommending face mask use by well persons in community settings as a means of avoiding infection during a pandemic. For community NPIs, one topic for additional study involves gathering empirical data on social mixing patterns in schools and community settings. These data can be used to create high-fidelity, high-resolution mathematical models of virus transmission in these settings to facilitate data-driven evaluations of different social distancing measures. Another area of research for community NPIs involves assessing the potential secondary consequences (e.g., missed work) of select community-level measures (e.g., school closures) for families, communities, and society to assess the economic effects of these measures. For environmental NPIs, additional research is needed to better understand surface contamination (e.g., which types of surfaces are more likely to be contaminated with influenza viruses) and identify situations in which surface cleaning should be emphasized (e.g., in households with confirmed influenza cases versus in healthy households). Additional information about NPI research gaps is available (supplementary Chapter 3 https://stacks.cdc.gov/view/cdc/44313).

## Conclusion

The 2009 H1N1 pandemic provided an opportunity to test, in practice, the key concepts of NPIs in mitigating the impact of an influenza pandemic, just 2 years after the publication of the 2007 guidance. As the experience from 2009 has shown, NPIs can be a critical component of pandemic influenza mitigation. Although well-matched pandemic vaccines remain the main tool in reducing the risk of acquiring infection and in controlling the spread of a pandemic virus, vaccines might not be widely available for up to 6 months after the emergence of a pandemic influenza virus, given current vaccine production technology. Furthermore, as during the 2009 H1N1 pandemic, antiviral medications might be prioritized for treatment but not used for widespread chemoprophylaxis because of concerns about antiviral resistance and limited stockpiles of antiviral medications. Therefore, NPIs might be the only prevention tools readily available for persons and communities to help slow transmission of an influenza virus during the initial stages of a pandemic. However, individual NPIs might be only partially effective in limiting community transmission when implemented alone. Thus, the most efficient implementation involves early, targeted, and layered use of multiple NPIs (https://www.cdc.gov/flu/pandemic-resources/planning-preparedness/community-mitigation.html). In addition, some community-level NPIs that potentially have the greatest epidemiologic effects on pandemic influenza virus transmission in communities, most notably school closures and dismissals, also are most likely to be associated with secondary (unwanted) consequences (*104*). Hence, prepandemic planning, including engaging communities in planning activities well ahead of the next pandemic, is critical to enable appropriate local decision-making during the early stages of a pandemic.

After the 2009 H1N1 pandemic, evidence on the effectiveness and feasibility of NPIs expanded substantially. A summary of the evidence in this 2017 update includes 2009 H1N1-related research (supplementary Appendix 5 https://stacks.cdc.gov/view/cdc/44314). However, knowledge gaps remain and should be addressed by future research. Further updates of these guidelines will be developed and issued when significant new information and evidence emerges about the effectiveness and feasibility of NPIs in mitigating the impact of pandemic influenza.

## Footnotes

* The updated 2017 planning guidelines do not address pandemic vaccine development and distribution, use of respirators in community or health care settings during a pandemic, or travel restrictions during a pandemic. Guidance and policies in these areas will be developed separately, as needed.

† In July 2009, ACIP recommended vaccination of several population groups, including children aged 6 months through 18 years because cases of H1N1 influenza had occurred in children who were in close contact with each other in school and child care settings (https://www.cdc.gov/h1n1flu/vaccination/acip.htm).

§ The pandemic mitigation framework proposed in the 2007 strategy was based on the early, targeted, and layered use of multiple NPIs. NPIs should be initiated early in a pandemic before local epidemics grow exponentially, be targeted toward those at the nexus of transmission (in affected areas where the novel virus circulates), and be layered together to reduce community transmission as much as possible. A list of NPIs that are recommended at all times and those that are reserved for pandemics is provided (Table 1).

¶ Influenza pandemics can range from mild to extreme in terms of rates of viral transmission and clinical severity, as described in the four prepandemic planning scenarios developed by CDC. A *very severe or extreme pandemic*, such as the 1918 pandemic, is characterized by high to very high transmissibility and clinical severity. A *severe pandemic,* such as the pandemics of 1957 and 1968, is characterized by high transmissibility and low to medium clinical severity. A *mild or moderate* *pandemic,* such as the 2009 pandemic, is characterized by low to medium transmissibility and clinical severity.

** Although many authorities use either 100°F or 100.4°F (37.8°C) as indicative of fever, this number can vary depending on factors such as the method of measurement and the age of the person. Therefore, other values for fever could be appropriate. CDC has public health recommendations that are based on the presence (or absence) of fever (i.e., persons’ temperature is not higher than their own normal temperature).

†† Guidance for caring for persons at home who have influenza symptoms is available at https://www.cdc.gov/flu/consumer/caring-for-someone.htm.

§§ Additional information about isolation and quarantine is available at https://www.cdc.gov/quarantine/QuarantineIsolation.html.

¶¶ If the incubation period for the next pandemic were shorter or longer than 3 days, CDC would amend the recommendation accordingly.

*** Additional information on the use of preemptive, coordinated school closures and dismissals of different durations is available (supplementary Chapter 3 https://stacks.cdc.gov/view/cdc/44313).

¶¶¶ Antimicrobial products registered for use against H1N1 influenza and other influenza A viruses on hard surfaces (https://archive.epa.gov/pesticides/oppad001/web/pdf/influenza-a-product-list.pdf).
